# Maritime-oriented foragers during the Late Pleistocene on the eastern costa del sol (Southeast Iberia): Cueva Victoria (Málaga, Spain)

**DOI:** 10.1016/j.heliyon.2022.e09548

**Published:** 2022-05-27

**Authors:** Esteban Álvarez-Fernández, J. Emili Aura Tortosa, Jesús F. Jordá Pardo, Ismael Palomero-Jiménez, Mª Teresa Aparicio, Lidia Cabello-Ligero, Pedro Cantalejo, Margarita Vadillo Conesa, Yolanda Carrión Marco, María del Mar Espejo, Mª José Fernández-Gómez, Naroa García-Ibaibarriaga, Adolfo Maestro, Ricard Marlasca, F. Javier Martín-Vallejo, Xavier Murelaga, Manuel Pérez-Ripoll

**Affiliations:** a Departamento de Prehistoria, Historia Antigua y Arqueología, Universidad de Salamanca, Calle Cerrada de Serranos s/n, 37002, Salamanca, Spain; b Grupo de Investigador Reconocido PREHUSAL, Universidad de Salamanca, Spain; c Departament de Prehistòria Arqueologia i Història Antiga-PREMEDOC, Universitat de València, Avda. Blasco Ibañez 28, E-46010, València, Spain; d Laboratorio de Estudios Paleolíticos, Departamento de Prehistoria y Arqueología, Facultad de Geografía e Historia, Universidad Nacional de Educación a Distancia, Paseo Senda del Rey 7, 28040, Madrid, Spain; e Museo Nacional de Ciencias Naturales, CSIC, C. José Gutiérrez Abascal, 2, 28006, Madrid, Spain; f Cave of Ardales, Av. de Málaga, 1, 29550, Ardales, Málaga, Spain; g Museo Municipal de la Historia y las Tradiciones de Ardales, Cueva de Ardales, Ayuntamiento de Ardales, Avda. de Málaga, 1, 29550, Ardales, Málaga, Spain; h Departamento de Estadística, Facultad de Medicina, Universidad de Salamanca, Campus Miguel de Unamuno, 37007, Salamanca, Spain; i Departamento de Geografía, Prehistoria y Arqueología, Facultad de Letras, Universidad del País Vasco UPV/EHU, C. Tomás y Valiente s/n, 01006, Vitoria-Gasteiz, Spain; j Department of Geoscientific Research and Prospective, IGME, Calle Calera 1., E-28760, Tres Cantos, Madrid, Spain; k Posidonia SL, Eivissa, Ricard, Spain; l Departamento de Geología, Facultad de Ciencia y Tecnología, Departamento de Estratigrafía y Paleontología, Universidad del País Vasco UPV/EHU, Apartado 644, 48080, Bilbao, Spain

**Keywords:** Coastal exploitation, Animal resources, Upper magdalenian, Mediterranean Iberia

## Abstract

The Mediterranean coast of Spain is marked by several clusters of Palaeolithic sites: to the south of the Pyrenees, in the area around the Ebro River, in the central part, and on the south coast, one of the southernmost regions in Europe. The number of sites is small compared with northern Iberia, but like that region, the Palaeolithic occupations are accompanied by several rock art ensembles. The archaeological material (both biotic and abiotic resources) and radiocarbon dates presented here were obtained during archaeological fieldwork of professor J. Fortea in the Late Pleistocene deposits in Cueva Victoria, located near the modern coastline and about 150 km north of the Strait of Gibraltar. In the three occupation phases, marine resources were acquired by shell-fishing (focusing almost exclusively on the clam *Ruditapes decussatus*), fishing, and the use of beached marine mammals. This contrasts with the limited data about the exploitation of terrestrial resources by hunting and gathering animals and plants. The study is completed by the study of artefacts (lithic and bone industry and objects of adornment) that help to understand the subsistence strategies of the cave occupants and enable a comparison with other groups inhabiting the Mediterranean coasts of the Iberian Peninsula during Greenland Interstadial 1, between *ca*. 15.1 and 13.6 cal BP.

## 1 Introduction

The exploitation of aquatic resources is an indicator of the ability of our genus to obtain a wide and flexible diet in the course of our biological and cultural evolution (Ungar et al., 2006; Archer et al., 2014; Cunnane and Stewart, 2010). It is a quality that is not exclusive to modern humans (Marean et al., 2007; Marean, 2014; Brown et al., 2011) as recently debated once more (Zilhão et al., 2020) and is currently regarded as a significant factor in studies on human evolution (Tattersall, 2014). This process has followed diverse lines, with regional variations observed in the use of these environments and raw materials, especially marine resources. The differences have been linked to geographic, ecological and social factors, but also regional to research traditions (Erlandson, 2001; Bailey and Milner, 2002).

Southern Iberia is a key region to study changes in the use of coastal areas and marine resources by prehistoric foragers in the western Mediterranean (Colonese et al., 2011; Álvarez-Fernández et al., 2014; Aura et al., 2016). Changes in the visibility of this type of exploitation/adaptation have been related to the morphology of the continental margins, which has allowed the conservation of littoral sites (Erlandson, 2001; Bailey, 2004). The available data for the Mediterranean region display significant differences between sectors (Aura et al., 2016; Román et al., 2020). The characteristics of the continental margin and the rise in mean sea level since the LGM are decisive factors in the explanation of regional variations in the use of marine resources (Jordá Pardo et al., 2011; Maestro et al., 2013).

The visibility and quantities of marine resources at coastal sites in southern Iberian, between the Gulf of Almeria and Gibraltar, increase at the end of the Pleistocene. Cueva de Nerja has provided much of the regional information (Aura et al., 2002, 2013; Jordá Pardo et al., 2011, 2016; Álvarez-Fernández et al., 2014; Morales-Pérez et al., 2020), to which the results of the present study of Cueva Victoria (Rincón de la Victoria, Malaga) can be added. This site forms part of the cluster of archaeological locations on the Bay of Malaga, known in the literature as the El Cantal/La Cala del Moral group (Cueva de Hoyo de la Mina, Cueva del Higuerón or del Suizo, Cueva Victoria and Humo/La Araña Complex).

The aim of the present study is therefore to present the archaeological record in Cueva Victoria. The results support a discussion of taphonomic, techno-economic and functional aspects not documented previously and which enlarge our knowledge of coastal sites in southern Iberian, which is greatly conditioned by the data obtained in Cueva de Nerja. Their regional contextualisation suggests hypotheses about changes in the mobility of human groups and how these affected symbolic representations, bearing in mind the concentration of aquatic and marine motifs in the regional Palaeolithic art: birds, fish and mammals (Aura et al., 2016).

The present study also possesses historiographic interest. The documentation and remains that have been studied come from the archaeological excavation carried out in Cueva Victoria by Professor F. Javier Fortea Pérez in 1972 and are deposited in the Department of Prehistory, Ancient History and Archaeology in the University of Salamanca. This excavation revealed the stratigraphic basis for the definition of the Mediterranean upper Magdalenian and established its later transformation into the microlaminar Epipalaeolithic (Fortea, 1973). The study of the lithic and bone industry in the Mediterranean Magdalenian allows the data from Cueva Victoria to be inserted in a wider context, in which this regional cluster is unique as the southernmost in Europe, displaying a systematic exploitation of marine resources (Aura, 1995; Aura et al., 2009). The integral study of the remains from the 1972 excavation was postponed at that time and has only been possible now.

## 2 Cueva Victoria

### 2.1 Geographical and geological settings

Cueva Victoria is located in the town of Rincón de la Victoria (Málaga, Spain). Its UTM coordinates are X = 383.963; Y = 4.064.427 (ETRS89, Zone 30). It is at about 70m above sea level (asl), and 580m in a straight line from the sea-cliffs at El Cantal Cape. The modern entrance of the cave is in an area of waste ground on La Esmeralda Hill, which is surrounded by housing estates, about 2km to the east of Totalán creek.

**Figure 1:**
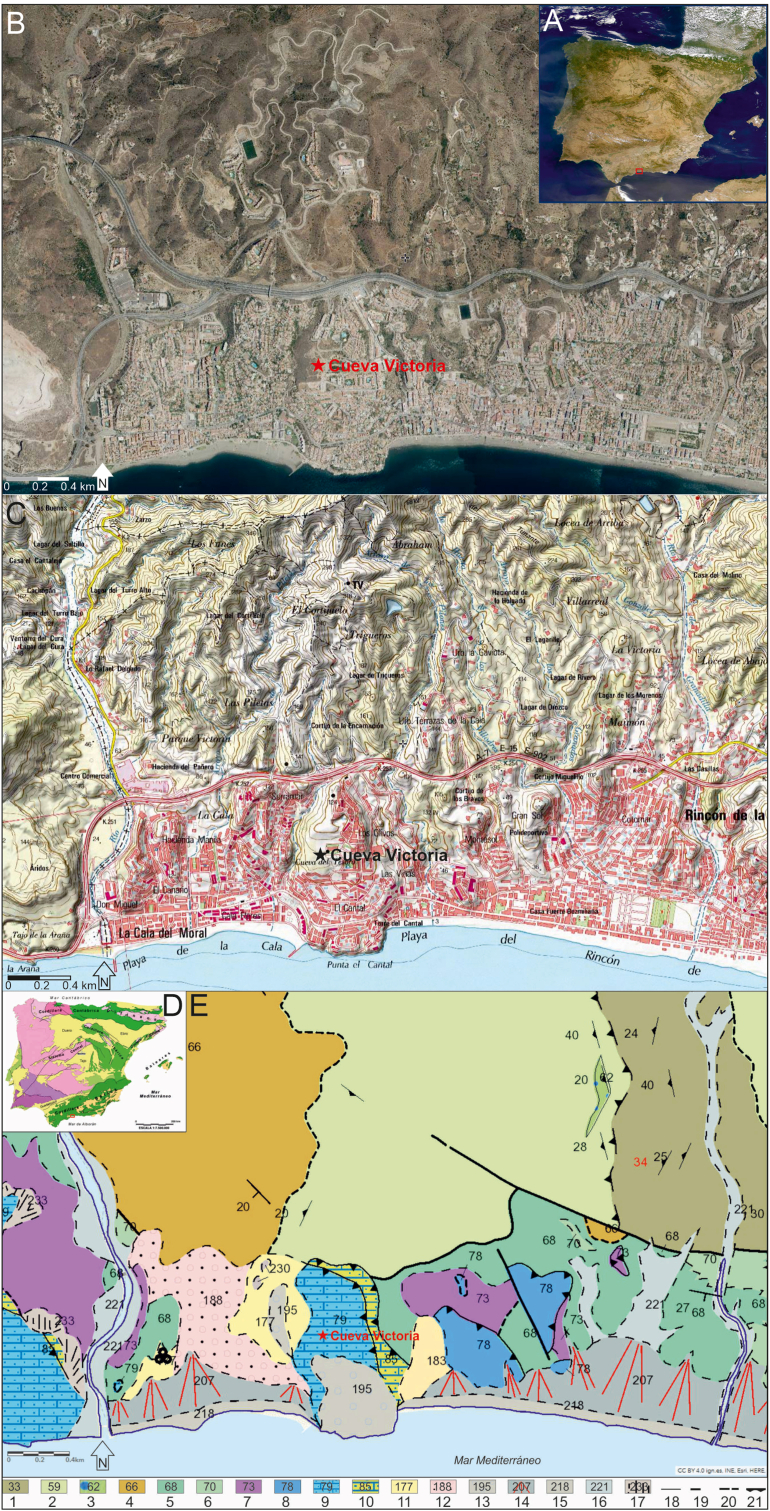
Location of Cueva Victoria in Iberia (A, Google Earth image), on an orthophoto (B) and on a map (C) (taken from SigPac: https://sigpac.mapa.gob.es/fega/visor/). (C) Geological setting of Cueva Victoria in the context of the Iberian Peninsula and (D) geological map of the area of the cave (taken from the Continuous Geological map of Spain scale 1/50.000 del IGME http://info.igme.es/visorweb/). Legend: Undifferentiated Palaeozoic, 1 schists, quartzites and calco-schists, 2 shales and greywackes, 3 conglomerates; Devonian, 4 turbiditic limestones; Carboniferous, 5 shales and greywackes, 6 conglomerates; Middle Triassic, 7 conglomerates, sandstones and clays; Lower Jurassic, 8 dolostone and marls; Middle Jurassic, 9 oolitic limestones and white massive limestones; Eocene (Ypresian), 10 fossiliferous detrital limestones, sandstones, calcarenites and marls; Miocene (Messinian), 11 blue marls; Upper Pliocene, 12 red clays, sands and conglomerates (alluvial fans); Quaternary, 13 marine terrace; Upper Pleistocene, 14 4th generation alluvial fans; Holocene, 15 beaches, 16 alluvials and valley bottoms, 17 dejection cones; 18 concordant contact, 19 fault, 20 discordant contact, 21 thrust.

Cueva Victoria is located in the Internal Zones of the Baetic Ranges, to be precise at the south-east end of the Maláguide Complex, in a small outcrop of Maláguide Jurassic limestone overlapping the Palaeozoic siliceous materials of the Alpujarride Complex (Figure 1) (Vera and Martín-Algarra, 2004). In the surroundings of the cave, these are represented by metamorphic and sedimentary rocks, such as graphitic schists, quartzites and calco-schists in the east, and slates, greywackes and conglomerates to the north and east. Devonian turbiditic limestone outcrops to the north-west, and Carboniferous shales, greywackes and conglomerates in the east. Middle Triassic conglomerates of quartz, sandstone and red clay; lower Jurassic dolostones with interbedded shales; and middle Jurassic oolitic limestones and massive white limestones overlie the Carboniferous materials. The Cueva Victoria system was formed precisely in those Jurassic limestones. A narrow band of Ypresian (lower Eocene) detritic limestones, sandstones, calcarenites and shales are located between the limestones and the Carboniferous slates. Messinian blue marls, corresponding to basin sediments, are discordant on these and the Palaeozoic materials to the west of the carbonate massif with the cave and discordant on these and the Palaeozoic materials, deposits of red clays, sands and conglomerates correspond to alluvial fan systems developed during the Upper Pliocene. Discordant on the Messinian marls are the deposits of one of the Pleistocene marine terraces whose distal end outcrops at 40m and the apical one at 80m asl, whose altitudinal development corresponds to those located at 50–60m in the La Araña area (Durán and Soria, 1989; Lhénaff, 1967; Lario et al., 1993 and 1998) and between 50-60m and 75–90m in the same area (Ferré et al., 2003).

These geological strata have formed a series of elongated hills perpendicular to the coast and separated by deep ravines. At their exits, surrounding the distal ends of the interfluvial land, alluvial fan deposits of fourth generation, of Upper Pleistocene age, have formed a strip parallel to the coast, between it and the hills. The ravine bottoms are covered by Holocene detritical deposits of alluvial character and to the west, Holocene alluvial fans have formed over the Triassic and Jurassic materials. Finally, the whole coastline, apart from El Cantal cape, is surrounded by the sand, gravel and pebble deposits that form the beaches.

**Figure 2:**
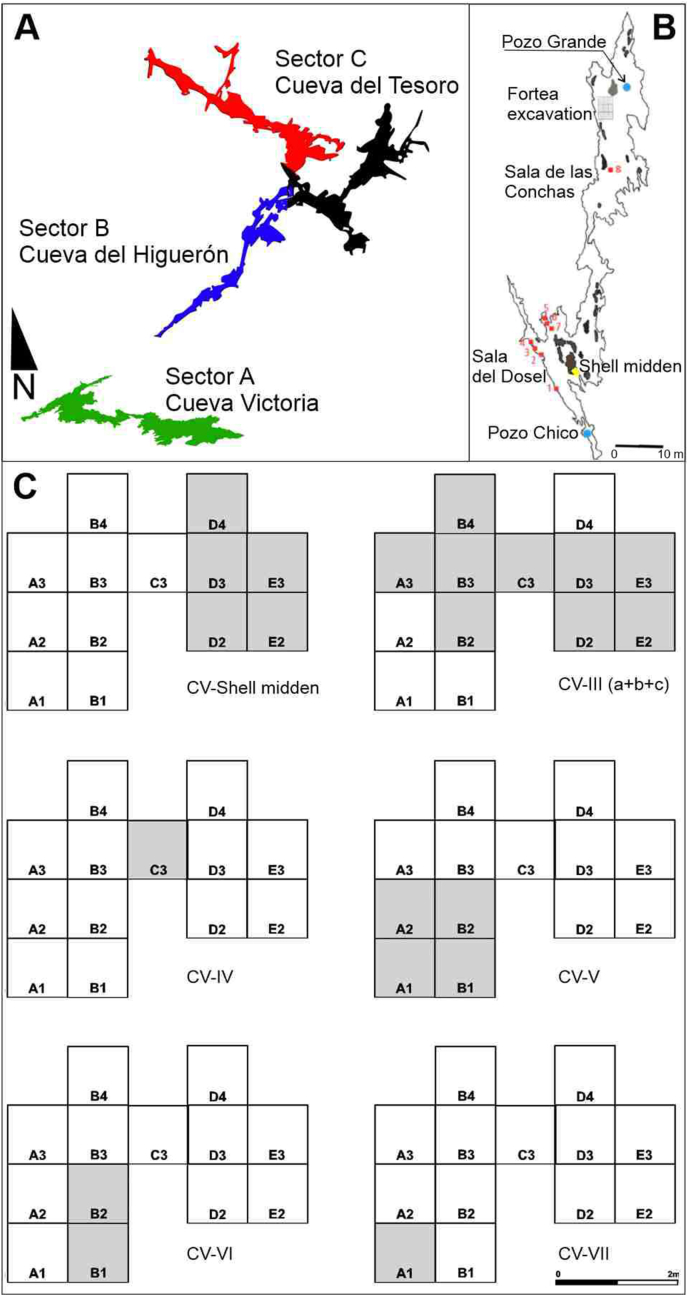
A: Karstic system of Cueva Victoria-El Higuerón-El Tesoro. B: Cueva Victoria (numbers in red indicate rock art panels). C: 1972 excavations of Professor Fortea with indication of the excavated squares and levels with archaeological evidence.

Cueva Victoria forms part of the El Cantal karstic system, which consists of three interconnected sectors (Figure 2A): Sector A, Cueva Victoria; Sector B, Cueva de El Higuerón or El Suizo; and Sector C, Cueva del Tesoro. It is a cave, less than 100m long, with two entrance shafts. The current access is through the cave ceiling, protected by a concrete construction, down aluminium stairs. The so-called “Pozo Grande” enters the “Sala de las Conchas” (Figure 2B) while the “Pozo Chico” leads to “Sala del Dosel”. The three sectors/chambers make up a horizontal passage formed by water under pressure that, after base level descent, changed into vadose conditions allowing the growth of speleothems, many of which were eroded when the cave was occupied by sea water during the Quaternary (Durán, 1996). The end of the passage is filled by detritic sediment that forms the modern cave floor. Some of these sediments correspond to the archaeological deposit in the cave. In sum, it is a fossil mountain karst system, with an autochthonous source, in the Mediterranean area of the Baetic-Murcian region, according to the classification of Llopis Lladó (1970). El Cantal of Rincón de la Victoria karstic system does not appear on the Karst Map of Spain (Ayala et al., 1986) apart from an indication of the tourist cave Cueva del Tesoro and El Higuerón.

Besides Cueva Victoria, Cueva de El Higuerón or El Suizo also contains an important archaeological deposit, which was excavated in the early twentieth century (Such, 1920; Cantalejo et al., 2007). Next to El Cantal cave system, only 2km away towards the east, are located the prehistoric sites of La Araña (La Cala del Moral, Rincón de la Victoria), also with important prehistoric occupations (Ferré et al., 2003; Ramos, 2003).

### 2.2 History of research

Archaeological research was carried out at different times in the twentieth century. It was discovered by Señor Román in 1939, and shortly after the discovery Simeón Giménez Reyna and Carlos Rein Segura excavated the site in its so-called “Sala del Dosel”, in 1941 and 1946. They documented three levels (Cantalejo et al., 2007; Rubio, 1975): a first superficial level 50cm thick, with Neolithic archaeological remains; a second level about 20cm thick formed by an accumulation of ash and apparently archaeologically barren; and a third level, about 70cm thick, without pottery, but containing pebbles, flints and a large number of shells, among other remains. Giménez Reyna first described the Palaeolithic-Epipalaeolithic deposit in Cueva Victoria in 1946: “… until the bedrock in the cave is reached, there is fill 0.7m thick with earth, some hearths and many mollusc valves, a few chipped flints and some pebbles” [translation from Spanish by the authors, TSA]. A few lines later, he adds: “…the whole fill in the floor is formed by an astounding quantity of shells (…) whose remains concord with the finds at El Higuerón and in the bottom of Hoyo de la Mina” [TSA] (Giménez Reyna, 1946: 30).

These first results agree with known information about the other sites in El Cantal. Some decades later, two complete flat barbed points/harpoons were cited by E. Ripoll (1970) and described by J. Fortea (1986). These bone artefacts possess a single row of barbs and are engraved, one with motifs formed by double angles and incisions transversal to the axis of the harpoon and the other with a zigzag that surrounded the axis.

The next reference to the stratigraphy in Cueva Victoria is in J. Fortea's PhD Dissertation (1973), in the discussion of the Late Palaeolithic-Epipalaeolithic sequence in Mediterranean Iberia. After studying the remains from Hoyo de la Mina and the El Cantal sites, he concludes that “… also in Cueva Victoria there seems to be a sequence: upper Magdalenian-Epigravettian-Shell-midden Facies, with an industry of chipped pebbles and broken cobble-stones, as found in the excavations carried out in summer 1972” [TSA] (Fortea, 1973: 320). This is the first explicit use of the term “shell-midden” to refer to a sedimentary and archaeological unit in the south of the Iberian Peninsula (Aura et al., 2013).

At the beginning of this century, work was carried out to protect and clean the cave (Márquez and Sanchidrián, 2006; Cantalejo et al., 2007). This work made it possible to close the access to the cave. The good condition of the area of the cave excavated by J. Fortea (Sala de las Conchas) was attested and evidence of occupations was documented in other parts of the site, where remains of said human occupations are preserved in sections. In that work, the collection of samples was not authorized, so only the conservation of those spaces was guaranteed and further analysis was not an option. At present, the cave is closed to the public. During the authorized visit made in 2018, we were able to recognize accumulations of shells, charcoal and bones in the profiles of those sections.

### 2.3 Excavation in the “Sala de las Conchas”

**Figure 3:**
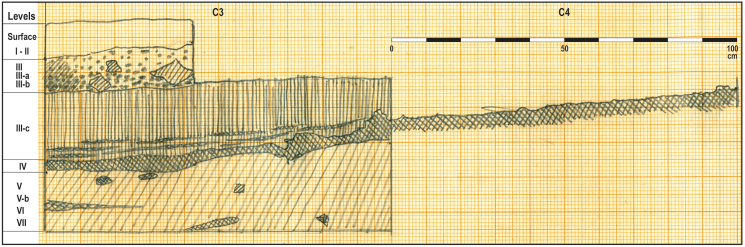
Schematic profile of Cueva Victori (“Sala de las Conchas”) sedimentary sequence.

**Figure 4:**
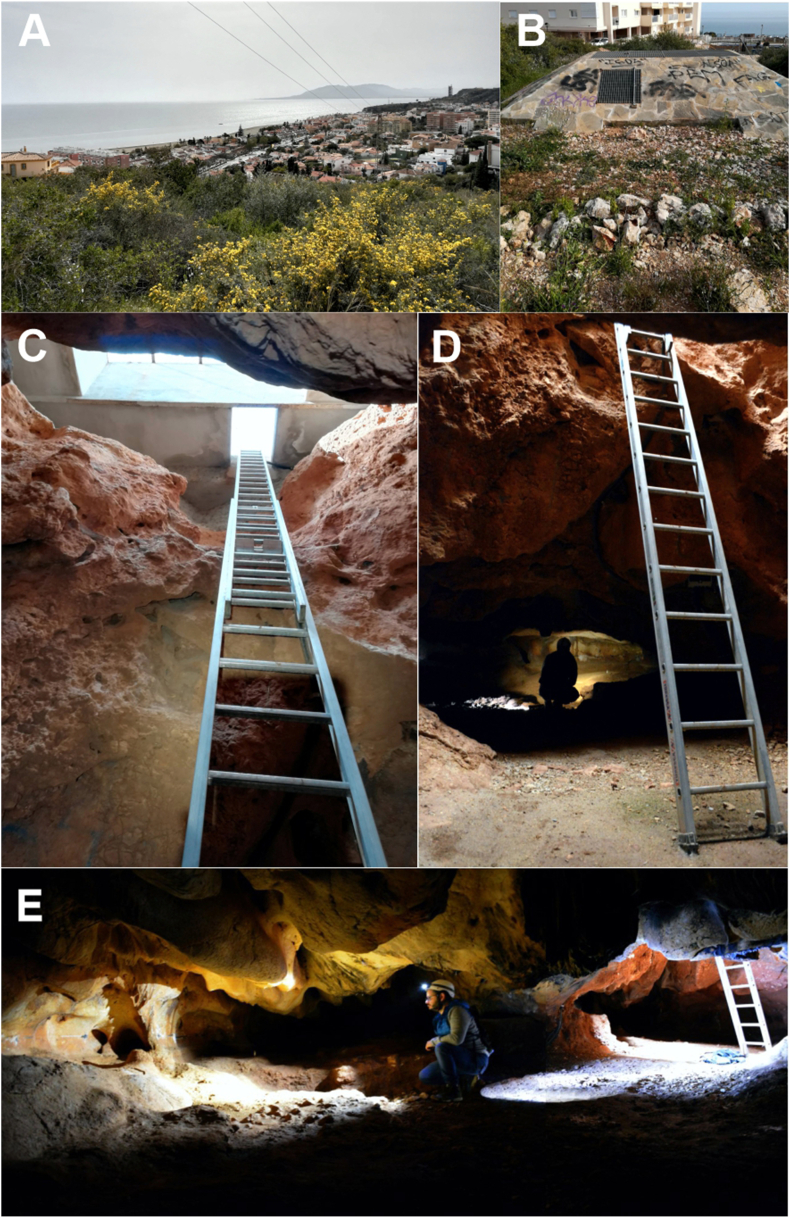
Cueva Victoria. A) View from the entrance to the “Sala de las Conchas”; B) Stone Wall protecting the entrance to “Pozo Grande”; C and D) Access to the “Sala de las Conchas”; E) Current view of the area excavated by J. Fortea in the “Sala de las Conchas”.

In 1972, F. Javier Fortea (1946–2009), at that time a lecturer at the University of Salamanca, excavated in the “Sala de las Conchas”. He included the results of this excavation very briefly in his PH D Dissertation and after studying the remains from Hoyo de la Mina and visiting the sites at La Cala del Moral, he noted the existence of Upper Magdalenian and “*Epigravettian*” occupations (Fortea, 1973: 320). However, he did not study or publish the archaeological remains found in his 1972 fieldwork. An initial reference to the sequence in Cueva Victoria identified three archaeological levels: upper Magdalenian, microlaminar Epipalaeolithic of Azilian derivation, and shell-midden with chipped pebbles (Fortea and Giménez, 1973). These remains were taken by F. J. Fortea to the Department of Prehistory, Ancient History and Archaeology at the University of Salamanca (Figures 3 and 4).

### 2.4 Stratigraphy and archaeological sequence: J. Fortea's excavation in the “Sala de las Conchas”

Professor Fortea's detailed 1972 excavation logbook and the information on the labels attached to the archaeological materials from the excavation have been used to reconstruct the stratigraphy in the sector of Cueva Victoria in which he carried out his fieldwork between the 5th and 30th of August (Figure 4). This sector was in the semi-shade zone in the main passage, below the “Pozo Grande”, known as “Sala de las Conchas”. In the part of the site that he called Ω, Fortea excavated 14 m^2^, in 1 × 1m squares. He describes a stratigraphic section between the modern floor level and the speleothem at its base. With this information (Figure 2C), the stratigraphy would consist from top to bottom as is represented in Table 1. The total excavated depth reached would be between 60 and 75 cm.

**Table 1:** Stratigraphic sequence at Cueva Victoria.

LEVEL	THICKNESS	DESCRIPTION	OTHER REMARKS
0	4cm	flowstone on which stratigraphic columns have formed	
I	6cm	highly carbonated clay	On the labels attached to the archaeological remains, he describes a “Surface Level” in Squares B2 and B4 as “earth accumulated at the entrance”.
II	2cm	thin flowstone layer	Excavated in Squares D2, D3, D4, E3 and E2. On the labels it is named “Shell-midden
III	16cm	clay with thin and discontinuous layers of flowstone in its interior	In the C-2/C-3 frontal-posterior section, he associated archaeological evidence and attributions to the described lithostratigraphic levels. Thus, Levels IIIa and IIIb correspond to what he called a “shell-midden” and Level IIIc to the Epipalaeolithic. Therefore, it seems clear that this was a sequence of cave fill with clay deposits cemented by calcium carbonate to a greater or lesser degree alternating with layers of flowstone. Some of the clay deposits contained clasts and looked like breccia. Level III apparently corresponded to a shell-midden. The labels on the archaeological materials show that in Squares B3, C3 and D2 it was classified as “Level III”; in Squares B2 and E3 as “Level IIIb”; in Squares A3, A5 and D3 as “Level IIIc”; and in Squares C4, D3, E3 and E4 as “Level III Epipalaeolithic”. Finally, in Square B4 he excavated a spit ascribed to “Level III in contact with the Surface Level” and another one described as “Level III Epipalaeolithic”.
IV	10cm	thick flowstone layer with interbedded clay and clasts with the appearance of breccia	The only archaeological remains come from Square C3
V	18 cm	similar clay to that of Level III, with some coarse elements	In Squares A1 and B1, Level V was divided into three sublevels: an upper level, Va, formed by “clayey sands, very cemented” by calcite; a middle one, level Vb, with “looser and darker clayey sands” and a lower one, Vc, similar to the previous sublevel but lighter in colour. In Square A1, a layer of flowstone was beneath Level V. In Square B1, he described a “layer of stones” that he did not consider a hut floor and instead attributed to “a floor with some habitation”, in which the stones seem “to have been placed both mechanically and by the transit of humans”. This layer of stones was located generally at the top of level Vb, as can be seen in the drawing of the B1/B2 frontal-posterior section. With the archaeological materials, labels indicate that “Level V/Vb between stones” was excavated in Squares A1, A2, B1 and B2, whereas the remains from “Level Vb/Vc” come from Squares A1, A2 and B1, and those from “Level Vb-VI” are only ascribed to Square A2
VI	1 cm	loose clay	It was excavated in Squares B1 and B2 according to the labels on the archaeological remains
VII	20–30 cm	flowstone layer	The only archaeological remains come from Square A1
VII "under the flowstone"	10 cm	very calcified layer	In the A1/A3 frontal-anterior section, beneath Level VII, which in the drawing of the section he called “2nd flowstone”. Excavated only in Square A1

Based on the 1972 excavation logbook and information obtained about the archaeological levels, J. Fortea's stratigraphic sequence can be grouped into three main archaeological phases. The option of determining their internal dynamic is difficult with the information currently available.

- **Phase A**: this corresponds to the Surface unit and Level CV-I separated by a layer of flowstone (II) and with no archaeological remains.
- **Phase B**: this corresponds to the excavation units described as a shell-midden (= Levels III, IIIa and IIIb shell-midden and IIIc. The documented archaeological materials can be grouped partly in CV-shellmidden and another part in CV-III.
- **Phase B/C:** level CV-IV is a layer of flowstone containing few archaeological and bioarchaeological remains. The date that has been obtained associates it most closely with Phase C, but without any conclusive evidence.
- **Phase C**: this corresponds to all the levels beneath the flowstone. The materials belong to levels CV-V, CV-Vb-VI, CV-VI and CV-VII.

This triple division takes into account the general dynamic of the formation of the deposit, the stratigraphic data and the archaeological materials found in each level. At a stratigraphic level, the identification of quite thick flowstone layers suggests extensive and iterated occupation cycles.

The results of the radiocarbon determinations given below identify two episodes, with an intermediate date that does not correspond to its stratigraphic position. Therefore, while they provide coherent points of reference for the older and more recent occupations, there are serious doubts about the intermediate date.

The lithic and bone industry does not allow the three phases to be recognised and separated; firstly because of the small number of remains and secondly because at a regional scale, the techno-economic traits of the Upper Magdalenian, final Magdalenian and Epipalaeolithic are very similar (Aura, 1995; Aura et al., 2013, 2020). It is not feasible to divide this internal dynamic with the information currently available.

### 2.5 Radiocarbon and chronostratigraphy

Table 2 displays the radiocarbon data of three marine shell samples (superficial level, Level IIIc and Level Vc) analysed by the ORAU. All three come from the same square (D4). They have been calibrated with the IntCal 13 (Reimer et al., 2020) and CalPal 2019 Hulu (Weninger and Jöris 2008) curves, both included in the CalPal programme (version 2020.3; Weninger and Jöris 2004). The table gives the calibrated dates corresponding to the intervals centred on the probability distribution of the true calibrated date with a total probability of 95% (at 2σ), and expressed as dates cal BP, where 0 = AD 1950.

**Table 2:** Samples from J. Fortea's excavation in Cueva Victoria and the radiocarbon dates obtained in the ORAU. Dates calibrated with the IntCal 20 (Reimer et al., 2020) and CalPal 2019 Hulu (Weninger and Jöris 2008) curves, both included in the CalPal programme (version 2020.3) (Weninger and Jöris 2004).

Square	SU	Sample	Procedure	Lab. ID.	Date BP	SD	D13C	CalAge p (95%) cal BP (0 = AD1950)		AU
								INTCAL20	CalPal 2019 Hulu	
D4	CV-Superficial	Burnt *R. decussatus shell*	14C AMS	OxA-33464	11,875	45	1.3	13,850–13,570	13,870–13,550	PHASE A
D4	CV-IIIc	Burnt *R. decussatus* shell	14C AMS	OxA-33465	12,510	55	(-) 1.1	15,140–14,340	15,060–14,420	PHASE B
D4	CV-Vc	*R. decussatus* shell	14C AMS	OxA-33466	12,405	55	1.7	15,000-14,120	14,880–14,160	PHASE C

**Figure 5:**
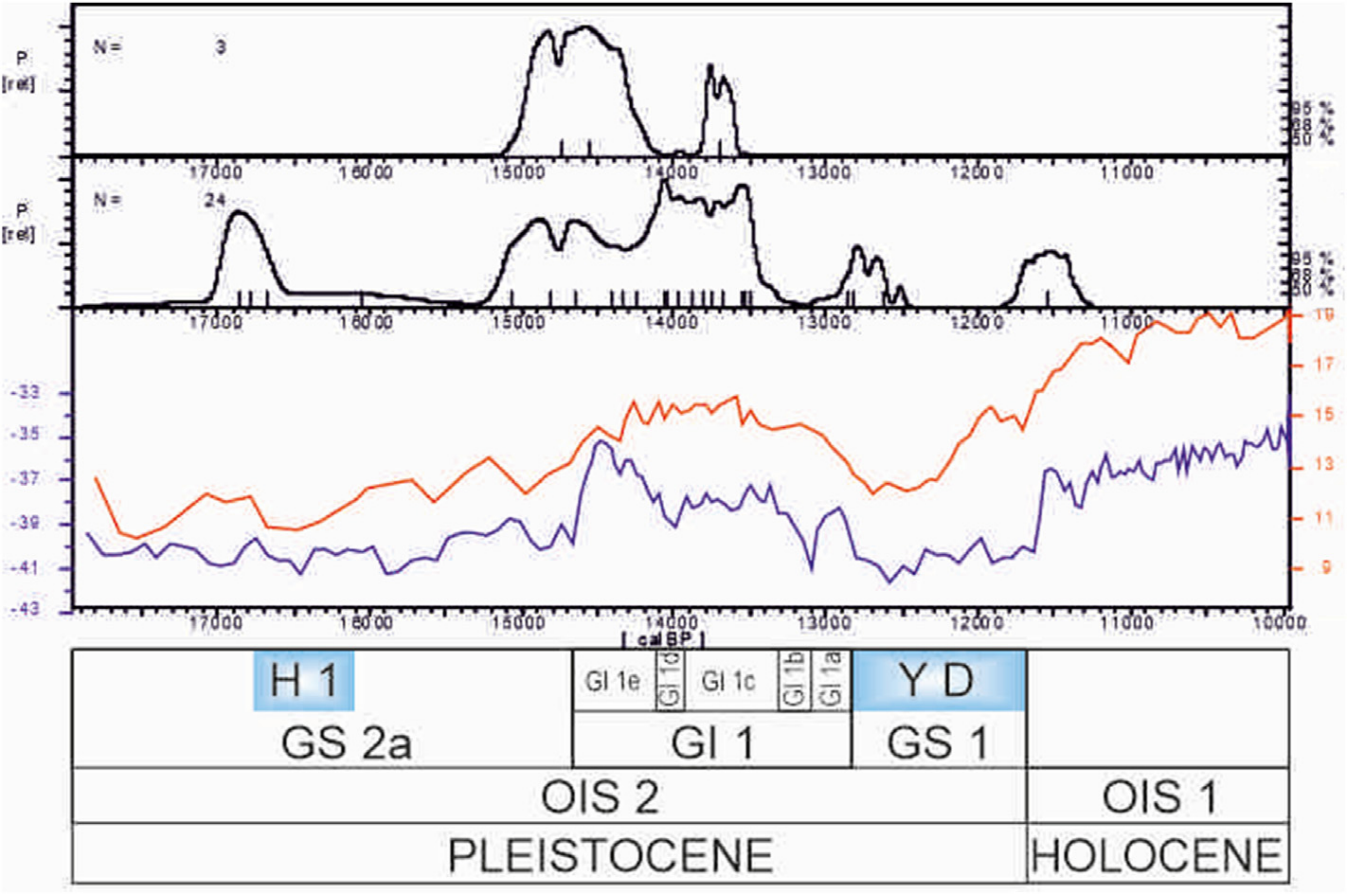
Cumulative probability curves of the calibrated dates from Cueva Victoria and other sites with Magdalenian levels on the Mediterranean coast in southern Iberia, with the IntCal 2020 calibration curve (Reimer et al., 2020), and their comparison with the curves for the δ18O GISP2 Hulu Age Model (Grootes et al., 1993; Meese et al., 1994; Wang et al., 2001) and Sea Surface Temperature MD-950243 for the Alborán Sea (Cacho et al., 1999, 2001), using the CalPal programme (version 2020.3; Weninger and Jöris 2004).

Figure 5 shows the cumulative probability curves of the three radiocarbon dates from Cueva Victoria and other coastal sites with Magdalenian levels in southern Iberia: Nerja (Jordá and Aura, 2008), Zafarraya (Barroso, 2003; Hoyo de la Mina (Ferrer et al., 2006), Humo 6 (Medved, 2013) and Gorham's Cave (Finlayson et al., 2006), using the CalPal 2019 Hulu calibration curve (Weninger and Jöris 2008), and the CalPal programme (version 2020.3; Weninger and Jöris 2004). Additionally, in order to situate the human occupations in Cueva Victoria on the Late Upper Pleistocene chronostratigraphic scale currently in use, based on the Greenland Events (Björck et al., 1998), the two groups of radiocarbon dates (Cueva Victoria and Alboran Magdalenian) have been compared with the curves for the δ^18^O GISP2 Hulu Age Model (Grootes et al., 1993; Meese et al., 1994; Wang et al., 2001) and Sea Surface Temperature MD-950243 for the Alboran Sea (Cacho et al., 1999, 2001).

The cumulative probability curve for the calibrated dates at CV displays two modes: one corresponding to Samples OxA-33465 and OxA-33466, centred on the interval 15,140–14,120 cal BP, during the late GS 2a and early GI 1, or to be more precise, in the temperate GI 1e period. The other is represented by Sample OxA-33464, in the interval 13,850–13,570 cal BP, also in a temperate phase of GI 1, in this case GI 1c. In the first interval, the sea surface temperature (SST) in the Alboran Sea rose rapidly from 12° to 15.5 °C, whereas in the second interval, the SST remained stable at about 15.5 °C, compared with the later fall in the SST during the GS 1 or Younger Dryas. With 95% probability, the three dates describe an interval centred on 13,690 cal BP, over a period of 1,360 years.

## 3 Morphologial and palaeogeographical evolution of Málaga continental shelf

**Figure 6:**
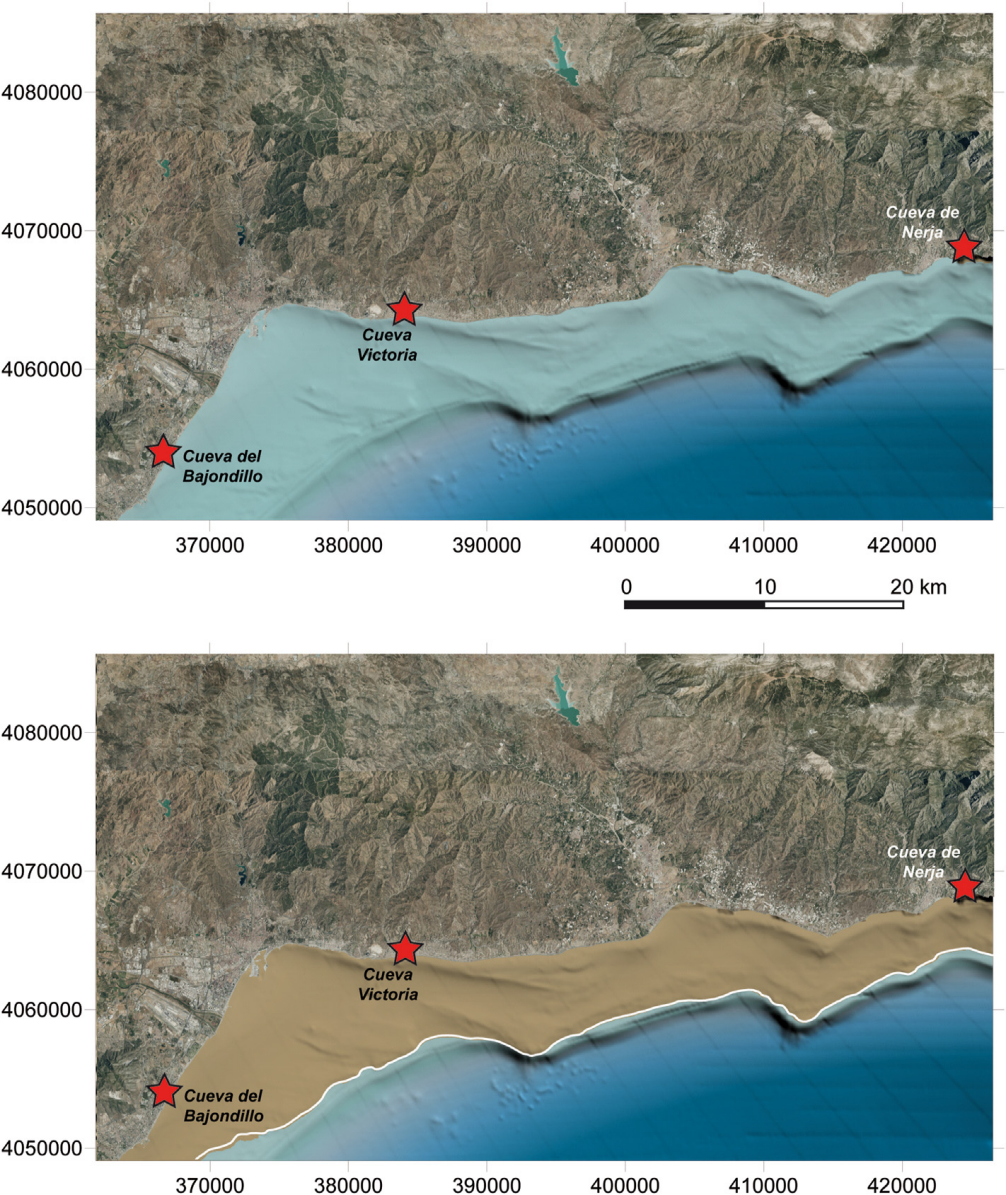
Reconstruction of the Malaga coastline between Benalmádena (W) and Nerja (E), marking the position of Cueva Victoria, during the period from 15.1 to 13.6 ka cal BP.

The palaeogeographical reconstruction of the sector around Cueva Victoria has been carried out by establishing the location of the coastline in the time interval when the site had human occupation, along a segment extending 77 km from the towns of Nerja to the east and Benalmádena to the west (Figure 6). In this sector of the Mediterranean coast, the continental shelf is narrow, between 4.2 and 11.7 km width, with the presence of sedimentary accumulations from the emerged areas due to the contributions from the Guadalhorce, Vélez, Torrox and Chillar rivers. Three sectors are differentiated in the continental shelf.

a) The inner-mid sector is characterized by gentle slopes with a mean gradient of 2° (Hernández-Molina et al., 1994a), and by sedimentary bodies as an infralittoral prism and prodeltaic bodies related with the river mouths (De la Cruz et al., 1992; Hernández-Molina, 1993; Hernández-Molina et al., 1993, 1995; Fernández-Salas, 1996, 2008; Lobo et al., 2006; Bárcenas et al., 2009; Bárcenas, 2013). These prodeltaic deposits have steep slopes, both in the foresets and bottomsets, and the offlap-breaks are more abrupt and shallower than in most Mediterranean prodeltas (Lobo et al., 2006; Fernández-Salas, 2008; Bárcenas, 2013).
b) The mid-outer sector shows average slopes of around 0.5°. The main morphological features are submarine terraces and prograding sedimentary bodies that are sometimes related laterally. Submarine terraces are flat surfaces with a sharp slope break or steepness towards the basin. These terraces show good lateral continuity and are generally oriented parallel to the isobaths, and they are recognizable from 70 m depth, since in the shallowest areas they are fossilized under the Holocene sedimentary prism. Submarine terraces are interpreted as old coastal marine abrasion platforms, so they can be related to brief stops at sea level during the eustatic rise (Hernández-Molina et al., 1992, 1994b, 1996; Hernández-Molina, 1993). These terraces are located at very variable depths: -15, -17, -20, -22 to -26, -32 to -35, -37, -40, -50, -55, -60, -70, -80, -90, -100 and -110 m (Hernández-Molina et al., 1996), some of them coinciding with those defined by Flemming (1972) in the western Mediterranean. In this sector, small prograding sedimentary bodies with lobulated morphology have been observed, which are also laterally associated with terraces. These sedimentary bodies have been related to sea level stabilization periods of longer duration than those that generated the submarine terrace development (Duane et al., 1972; Sangree and Widmer, 1977; Belknap and Kraft, 1981), or even with brief intervals of falling sea level within the general sea-level rise trend (Hernández-Molina et al., 1994b). These small prograding bodies are located at -20, -33, -47, -55, -60, -73, -80 and -90 m depth.
c) The outer sector and the shelf edge area is constituted by the shelf break, which is located at approximately -110 m in average depth, with variations between -100 and -150 m depth. This sector is characterized by the development of sedimentary prograding bodies that have been interpreted as marginal deltas or sedimentary shelf edge wedges (Alonso et al., 1992).

**Figure 7:**
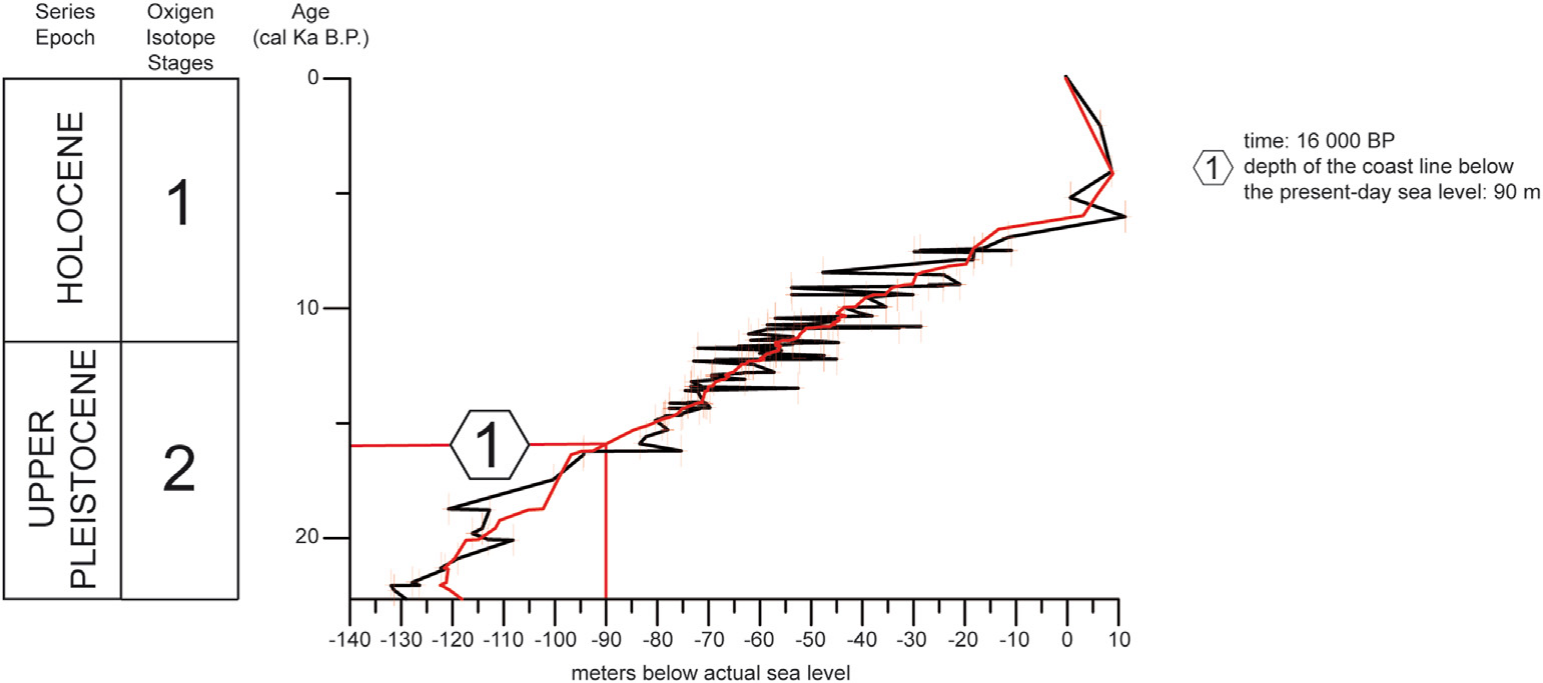
Variation in sea level in the last 22,000 years, indicating the period of study (based on the high-resolution relative sea level curves calibrated from the radiometric timescale SPECMAP; Thompson and Goldstein, 2006).

Paleogeography reconstruction of the coastline has covered the time interval between 13.6 and 15.1 ka BP, coinciding with the human occupation time interval established in Cueva Victoria (Figures 6 and 7). This time interval coincides with a stage of sea-level rise from the late Pleistocene to mid-Holocene (18–6.8 ka) (Shakelton, 1987; Berger et al., 1994; North Greenland Ice Core Project, 2004). This transgressive interval is related to the melting of continental ice sheets due to global climatic warming (Ruddiman and McIntyre, 1981). Sea level rose to 10 m depth below present sea level from 120-150 m depth below present sea level (Thompson and Goldstein, 2006). Significant development of depositional and erosional morphologies occurred on the continental shelf such as backstepping deposits, erosional terraces and transgressive erosional surfaces (Fernández-Salas et al., 2015). This process of sea-level rise was not continuous in time and interruptions have been observed established by short relative sea-level falls and standstills. In the analysed transgressive interval, the coastline was located at -73 m (Thompson and Goldstein, 2006) (Figure 7), coinciding with one of the terrace-small prograding sedimentary body levels established by Hernández-Molina et al. (1996). The coast was located between 3 and 8.4 km offshore from the present-day coastline (Figure 6) and about 6 km from Cueva Victoria. The emerged land surface near the study area was about 382 km^2^ larger (Figure 6) than at present-day.

## 4 Environment and exploitation of biotic resources

### 4.1 Anthracology

Only six fragments of charred wood <0.5mm in size were obtained in Cueva Victoria. Four of the fragments come from CV-III but lack a specific context. One of these is a conifer fragment (Figure 8.1 and Figure 8.2), which may belong to *Pinus nigra* and/or *Pinus sylvestris* (black pine and/or Scots pine) based on the possible presence of fenestriform pits in the cross-fields, although a radial section could not be obtained to confirm this (Figure 8.3). Two fragments of *Quercus* sp. (oak) have been identified by the presence of tangential parenchymal chains in cross-section (Figure 8.4), which are characteristic of that genus (Schweingruber, 1990). Finally, the other two fragments were identified as Fabaceae (legumes) (Figure 8.5 and Figure 8.6), one of them from an unknown provenience.

**Figure 8:**
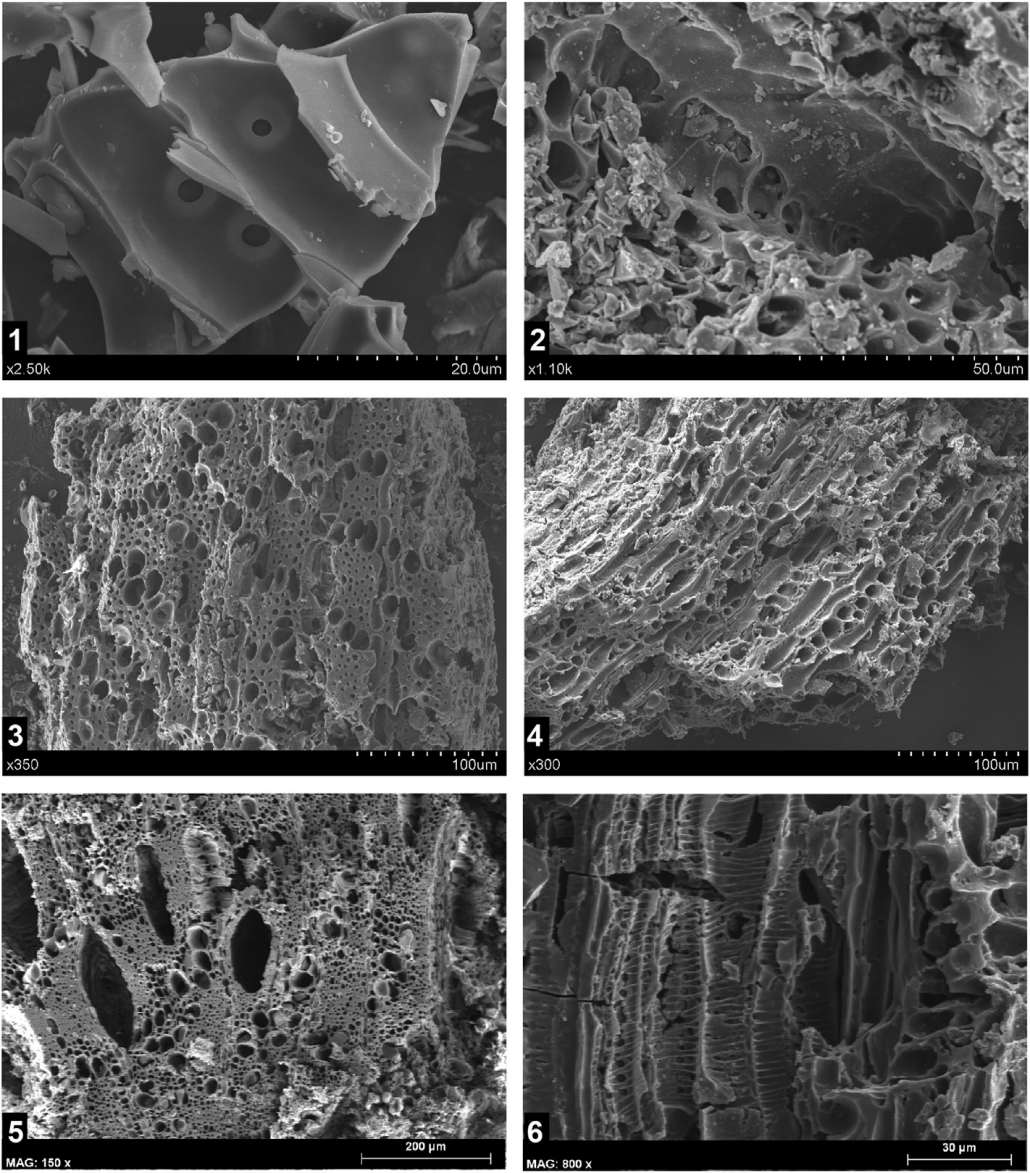
SEM photos of the identified charcoals. 1: Conifer, bordered pits; 2: Conifer, possible cross-field with fenestriform (“window-like”) pits; 3: Quercus sp., cross-section; 4: *Quercus* sp., tangential section; 5: Fabaceae, cross section; 6: Fabaceae, tangential section.

### 4.2 Archaeozoology

Table 3 summarises the faunal remains found in Professor Fortea's excavation in Cueva Victoria.

**Table 3:** Faunal remains documented in the sequence in Cueva Victoria.

	PHASE A		PHASE B						PHASE B/C		PHASE C							
	CV-Superficial		CV-Shell-Midden		CV-III (a + b)		CV-IIIc		CV-IV		CV-V		CV-Vb-VI		CV-VI		CV-VII	
	NR	MNI	NR	MNI	NR	MNI	NR	MNI	NR	MNI	NR	MNI	NR	MNI	NR	MNI	NR	MNI
TERRESTRIAL MAMMALS																		
*Equus ferus*	1	**1**																
*Bos/Bison*							1	**1**										
*Capra pyrenaica*			1	**1**	3	**1**	5	**2**	1	**1**	2	**1**						
*Cervus elaphus*	1	**1**	1	**1**	1	**1**	1	**1**	1	**1**								
*Sus scrofa*					2	**1**	2	**1**										
*Lynx pardina*			1	**1**			1	**1**										
*Felis sylvestris*			2	**1**			1	**1**										
*Vulpes vulpes*					1	**1**					2	**1**						
*Oryctolagus cuniculus*	8	**2**	100	**13**	110	**17**	72	**13**	9	**2**	65	**12**	33	**4**				
*Apodemus sp.*	4	**2**																
Subtotal	**14**	**6**	**106**	**18**	**117**	**21**	**83**	**20**	**11**	**4**	**69**	**14**	**33**	**4**				
MARINE MAMMALS																		
*Delphinus delphis*			1	**1**														
Subtotal			**1**	**1**														
BIRDS																		
Indet.			9	**1**	4	**1**	3	**1**										
Subtotal			**9**	**1**	**4**	**1**	**3**	**1**										
RODENTS																		
*Apodemus sp.*	4	**2**																
Subtotal	**4**	**2**																
REPTILIANS																		
*Lacertidae*			1	**1**														
Indet.			2	**1**														
Subtotal			**3**	**2**														
FISHES																		
*Sparus aurata*	1	**1**	4	**3**							2	**1**						
*Pagellus erythrinus*			1	**1**							1	**1**						
Diplodus sp.	1	**1**	1	**1**														
Sparidae Fam.			9				1	**1**										
*Mugilidae*			2	**1**							1	**1**						
*Anguilla anguilla*			1	**1**														
Indet.			30										1	**1**				
Subtotal	**2**	**2**	**48**	**7**			**1**	**1**			**4**	**3**	**1**	**1**				
CRUSTACEANS																		
*Tubicinella major*	1	1																
Carcinus sp.			4	2	2	2					2	1						
Subtotal	**1**	**1**	**4**	**2**	**2**	**2**					**2**	**1**						
MARINE MOLLUSCS																		
*Ruditapes decussatus*	307	96	2491	257	676	132	147	26	5	1	1197	164	67	7	161	13	11	1
Solen sp.	14	1	22	4	22	4	9	3			34	6			4	2	1	1
Cerastoderma sp.	1	1	8	4	10	3	3	2			13	4			2	1		
Pecten sp.	1	1	1	1	1	1	1	1			4	2	1	1				
*Gari depressa*	1	1																
Mytilus sp.									1	1								
Bivalve indet.			1	1							3	2						
Bittium sp.			1	1														
*Littorina obtusata*					3	3			1	1								
*Littorina sp.*							1	1										
*Tritia (T. pellucida/T. neritea)*			1	1														
Subtotal	**324**	**100**	**2525**	**269**	**712**	**143**	**161**	**33**	**7**	**3**	**1251**	**178**	**68**	**8**	**167**	**16**	**12**	**2**
CONTINENTAL MOLLUSCS																		
*Iberus alonensis*	11	**9**	24	**11**	63	**14**	31	**20**	7	**5**	14	**9**			4	**4**	6	**1**
*Iberus marmoratus*	1	**1**	14	**8**	6	**7**	6	**3**	2	**2**	4	**2**						
*Rumina decollata*	2	**2**									1	**1**						
Higromidae					1	**1**												
Gatropod indet.			2	**1**					1	**1**	3	**1**			7	**1**		
Subtotal	**14**	**12**	**40**	**20**	**70**	**22**	**37**	**23**	**10**	**8**	**23**	**13**			**11**	**5**	**6**	**1**

#### 4.2.1 Terrestrial fauna

##### 4.2.1.1 Microvertebrates

Small vertebrate remains are very scarce (n = 7, only located in CV-Superficial and CV-Shell Midden). They consist of disarticulated bone fragments and isolated teeth. They belong to Rodentia (*Apodemus* sp.) and Reptilia (Lacertidae).

##### 4.2.1.2 Large mammals

The sample consists of 433 remains, of which 411 have been identified taxonomically. Most of them are affected by calcareous concretions (*ca*. 90%), which has hampered the taphonomic study. The taxonomic range is wide. Rabbit (*Oryctolagus cuniculus*) clearly predominates in the assemblage, with 379 remains (*ca*. 92% of the total). Large mammals, i.e. aurochs (*Bos* sp.) and horses (*Equus caballus*), are present, but each with a single remain. Medium-sized mammals are represented by ibex (*Capra pyrenaica*, 12 remains), red deer (*Cervus elaphus*, 5 remains) and wild boar (*Sus scrofa*, 4 remains). Some small carnivores have also been identified (three remains of the wildcat *Felix sylvestris*, two of the lynx *Linx pardina* and three of the fox *Vulpex vulpes*).

As the osseous remains are affected by the precipitation of calcium carbonate, it has not been possible to identify butchery marks made with lithic tools. In the case of the rabbits, anthropic fractures, carnivore bites and digested bones have been documented. The digested bones and bites suggest that are large part of these animals were not consumed by humans. Breakages to extract bone marrow have been identified on a rib fragment and femur shaft fragment of ibex. The only aurochs bone is a humerus shaft fragment with a clearly anthropic fracture. Evidence of thermal alterations has only been recognised on five bones in the whole osseous assemblage.

Following the methodology for the ossification and size of rabbit bones (Sanchís, 2012), four age groups have been differentiated: adults, older than 10 months; sub-adults, 5–9 months; juveniles, 5-3 months; and very young, under 3 months. The analysed rests indicate that 38% of the individuals are adults, followed by sub-adults (33%), juveniles (16%) and very young (11%). This age structure suggests that the agents responsible for introducing the rabbits were both human and natural (small carnivores and raptors).

##### 4.2.1.3 Continental molluscs

The terrestrial mollusc assemblage from Cueva Victoria consists of 197 remains, of which a total MNI of 104 has been calculated. The two most common species, *Iberus alonensis* and *Iberus marmoratus*, form a large part of the record with 92.3% of the total MNI (70.2% and 22.1%, respectively). The assemblage is completed by three specimens of *Rumina decollata* and one of the Family Higromiidae. *Iberus alonensis* and *Iberus marmoratus* are the only edible species and would have been gathered as food. In contrast, *R. decollata* would have reached the cave fortuitously, adhered to other food, fuel or objects. This species is a scavenger and usually lives in places with detritus and waste. Only two of the *Iberus* sp. individuals have been altered by heat (2%, one from the “shell-midden” level and the other from Level III). All the continental mollusc taxa found in Cueva Victoria belong in the Mediterranean fauna that currently lives in the surroundings of the site and therefore, despite the scant assemblage, it may be proposed that the environmental characteristics at the time the deposit formed would not have been very different from the present conditions.

#### 4.2.2 Marine fauna (Figure 10)

##### 4.2.2.1 Molluscs

The shells of marine molluscs are the most abundant faunal remains throughout the sequence and are particularly numerous in CV-shell-midden, CV-III and CV-V (Figure 9.1 to 9.3). A total of 5,227 remains have been documented, from which a number of 752 individuals has been calculated. Bivalves dominate over gastropods in terms of both number of remains and MNI, with percentages >99% in both cases. Edible species predominate in the bivalves. The grooved carpet shell *Ruditapes decussatus* is the most abundant (96.8% in NR and 92.7% in MNI), followed at a distance by the razor shell *Solen* sp. (2.0% in NR and 2.8% in MNI) and the cockle *Cerastoderma* sp. (0.7% in NR and 2 % in MNI). The bivalve assemblage is completed by a few remains of *Pecten* sp., *Mytilus* sp. and *Gari depressa*. Gastropods are equally scarce in the sequence and have been identified as the species *Littorina obtusata*, *Tritia* sp. (*T. pellucida/T. neritea*), *Bittium* sp. and *Littorina* sp. These are very small species with no food value.

**Figure 9:**
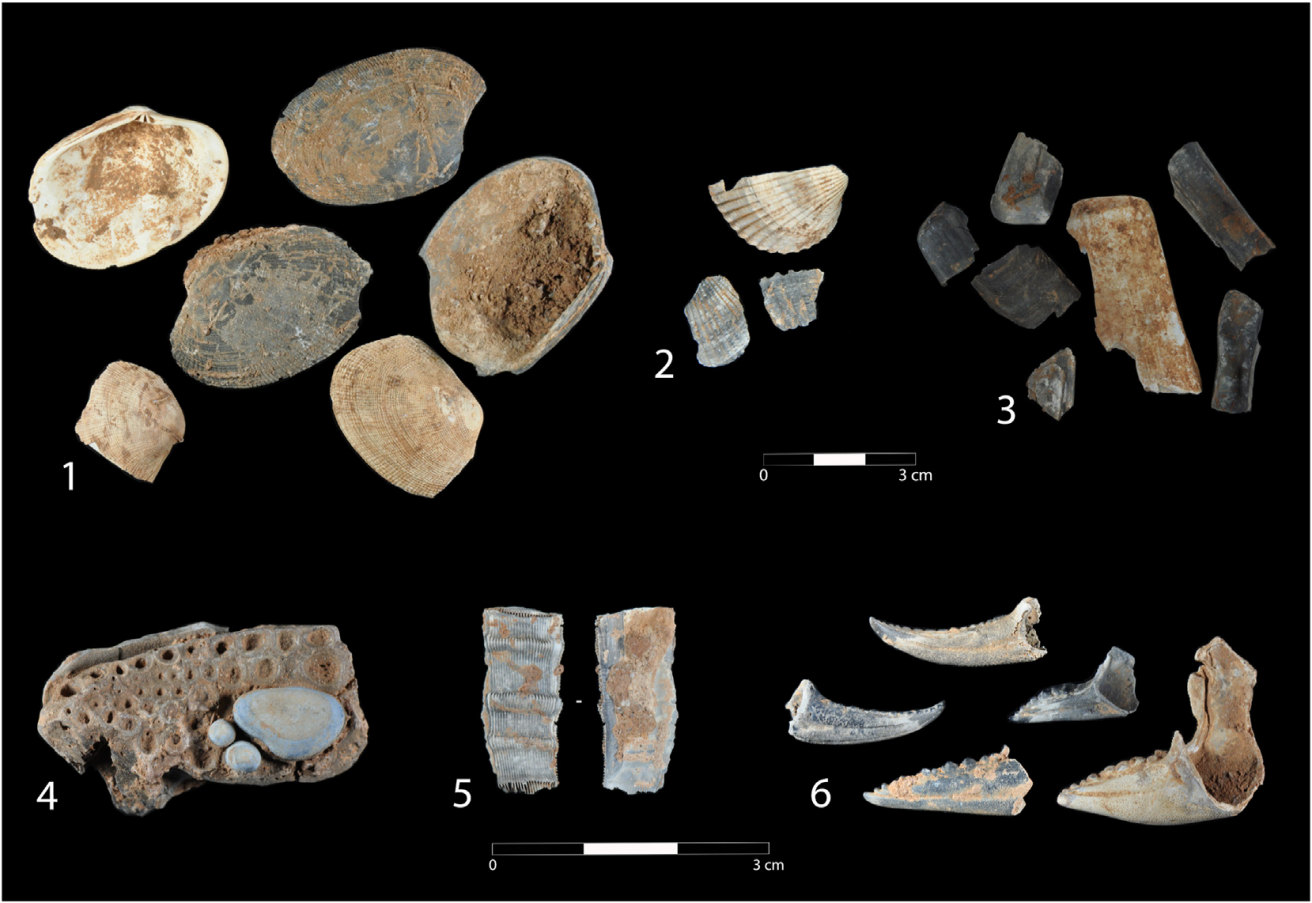
Mollusc (1-3), fish (4) and crustacean (5-6) remains in Cueva Victoria. 1. *Ruditapes decussatus* (CV-Vc, Phase C); 2 *Cerastoderma* sp. (CV-Vc, Phase C); 3. *Solen* sp. (CV-III, Phase B); 4. *Sparus aurata* (CV-Shell midden, Phase B); 5. *Tubicinella major* (CV-superficial, Phase A); 6. *Carcinus* sp. (CV-shell midden and CV-III, Phase B).

All these species except for *Littorina obtusata* are currently present on the coasts of the Alboran Sea. The three most abundant species live today in sandy or muddy substrates: *R. decussatus* on beaches and in estuaries, in the mid-shore and low shore zones, to 4m depth; *Solen* sp. on beaches and the continental shelf, in the mid-shore and low shore zones, to 20m depth; and *Cerastoderma* sp. in estuaries and marshes, in the low shore zone, to 2–3 m depth.

The first types of alterations to the shells that should be considered took place before they were gathered on the shore. In the case of the *Pecten* sp. shells, they would have been collected on a beach as they display marine erosion around their edges and perforations caused by shell-boring annelids (*Polydora* sp.). All the gastropod shells display signs of marine erosion on their surface which equally shows that they were gathered on beaches after the animal had died.

The most common alteration affecting the mollusc remains in Cueva Victoria is breakage. This is seen particularly on the razor shells, which are the most fragile. The index (NR/MNI)/100 has been used to calculate the intensity of the fragmentation in the different levels. The highest index is seen in CV-VI (0.122), and it deceases upwards in the stratigraphy to CV-superficial, with the lowest index (0.032). Breakage was caused by trampling and by the pressure of the sediment.

65.6% (n = 3,430) of the remains were altered by heat (571 of MNI: 75,9%). This is observed in all the levels. In the levels with the most remains, thermal alteration is distributed in the following way: CV- shell-midden, 89.6% NR and 76.3% of MNI; in CV-III, 62.8% NR and 44.6% of the MNI; and CV-V, 72.7% NR and 52.8% of the MNI. *L. obtusata* and *Tritia* sp. are the only species in which intentional anthropic modifications (perforations) have been documented.

All the shells are affected to a greater or lesser extent by the precipitation of calcium carbonate. Above all, in the shell-midden level, malacological remains are calcified to other archaeological remains: fragments of bones and stones, fish vertebrae, teeth and mandibles, etc. Only a few of the shells in the sequence have been affected by decalcification, causing corrosion of the external surface and the appearance of a whitish colour on their surface.

**Figure 10:**
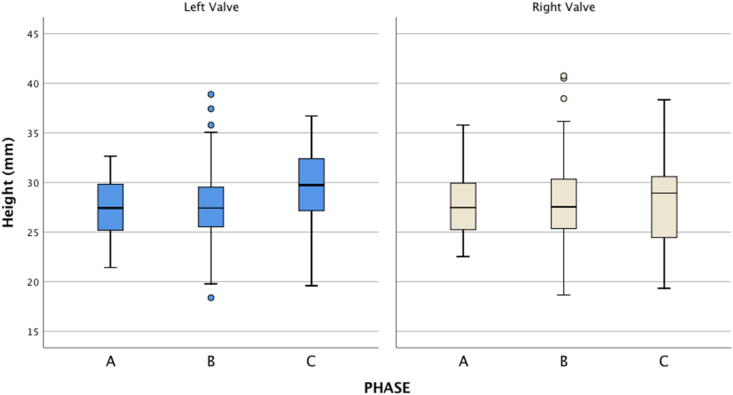
Box-plot for both *Ruditapes decussatus* valves in each phase. Left valve: Phase A (n = 58), Phase B (n = 137), Phase C (n = 39). Right valve: phase A (n = 59), phase B (n = 137), phase C (n = 39).

The biometric analysis of the shell sizes has considered the height of both left and right valves of the most abundant species (Figure 10), the grooved carpet shell *Ruditapes decussatus*, regarding the best conserved remains in the levels where it was most common. The results of descriptive statistics (n, mean ± S.D.) in the different phases, for the left valve are: Phase A (CV-superficial; n = 58, 27.53 ± 3.02), Phase B (CV-shell midden + CV-III + CV-IIIc; n = 137, 27.82 ± 3.52), Phase C (CV–V; n = 39, 29.46 ± 4.07), and for the right valve: Phase A (n = 59, 27.47 ± 3.04), Phase B (n = 137, 28.05 ± 3. 81), Phase C (n = 39, 29.47 ± 4.07).

Kolmogorov-Smirnov test were not significant for each combination of type of valve and phase (p-values>0.05). The ANOVA test were also not significant for the right valve (p-value = 0.603. Nevertheless, the ANOVA test was significant for the left valve (p-value = 0.017). The Bonferroni correction for multiple comparisons showed that the height was greater in Phase C than in Phase A and B (p-values = 0.024 and 0.031 respectively) but no differences were found between Phases A and B (p-value> 0.50).

##### 4.2.2.2 Crustaceans (crabs and acorn barnacle)

The crustaceans documented in the levels in Cueva Victoria belong to the Orders Decapoda and Sessilia. Eight remains of the former have been identified, corresponding to the *Carcinus* genus (Figure 9.6). These are the first extremities (pincers) from CV-shell-midden (four dactyls, two right and two left), from CV-III (two right propodi) and from CV-V (a right dactyl and a left propodus). They all come from adult individuals. Six of them are altered by heat. It has been estimated that they come from a minimum number of five individuals. They probably belong to the Mediterranean species *Carcinus aestuari*, which currently lives on the sandy substrates of the inter-tidal and low shore areas in the south-east of the Iberian Peninsula. The single acorn barnacle plate comes from CV-superficial. Based on its external and internal characteristics, it has been identified as a thermally-altered right lateral plate which is missing the opercular zone. It comes from the balanoid species *Tubicinella major* (Figure 9.5). The exoskeleton of this balanoid consists of six plates that fit together to form a cylinder. *Tubicinella major* is endemic to the whale *Eubalaena australis*.

##### 4.2.2.3 Fish

56 fish remains have been found, consisting of fragments of cranial bones, vertebrae and other bone fragments, most of which come from the CV-shell-midden (*ca*. 85.7%). Others were found in CV-superficial, and in CV-IIIc, CV-V and CV-V/VI. The identified remains belong mainly to sparids (n = 20) (Figure 9.3), among which cranial bones and vertebrae have been classified as gilt-head sea bream (*Sparus aurata*), and would have come from at least three specimens between 35 and 45cm in length.

Other identified remains are a cranial bone of a common pandora (*Pagellus erythrinus*) about 35cm long, and a vertebra of a *Diplodus* sp. about 40cm in length. Two vertebrae of mugilid 15cm and 40cm long and another of an eel about 70cm long have also been identified. In addition to the fragmentation of the remains, two of the vertebrae from the shell-midden layer and three mandible fragment are affected by heat. The predominance of the sparids suggests that fishing took place in littoral zones and coastal lagoons, mainly on rocky substrates.

##### 4.2.2.4 Birds

Only 16 bones of unidentified birds have been documented. Nine are in CV-shell-midden, four in CV-III and three in CV-IIIc. Their fragmented state and adhered calcium carbonate have not allowed a taxonomical identification.

##### 4.2.2.5 Marine mammals

**Figure 11:**
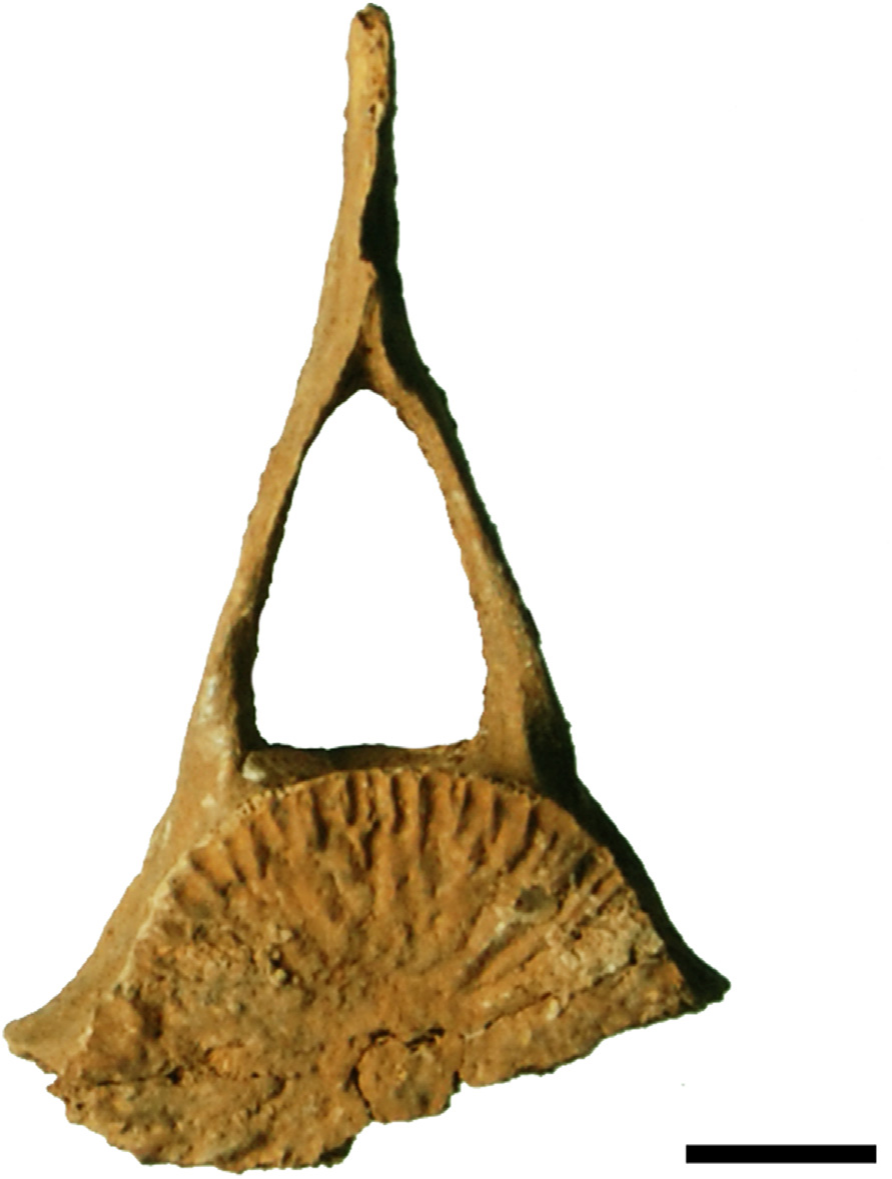
*Delphinus delphis* vertebra fragment from Cueva Victoria (CV-Shell Midden, Phase B).

The only evidence of a marine mammal is a vertebra fragment from a short-beaked common dolphin *Delphinus delphis*, which was found in CV-shell-midden (Figure 11). Precipitated calcium carbonate has hindered the observation of any possible anthropic alterations to the bone.

## 5 Bone and lithic technologies

### 5.1 Osseous assemblage (Figure 12)

Evidence of osseous industry have been documented on bone and shell remains.

#### 5.1.1 Fine points

Four implements have been documented in the osseous assemblage (Figure 12.1). Three come from Phase B (CV-shell-midden, CV-III and CV-IIIc) and the fourth from Phase B/C (CV-IV). They were all made from bones of small prey (bird or mammal) and in three cases they conserve part of the medullary canal. They vary a little in size, particularly in width and thickness (length between 18.0 and 23.4mm; width between 1.8 and 2.8mm; thickness between 1.1 and 1.5mm). The scraped and polished surfaces do not allow the determination of how the objects were obtained. Signs of a groove are seen in one case, but the possibility of percussion to extract fragments used as blanks cannot be ruled out. The four objects are altered by heat to different degrees (brown, black and grey colouring) and in one case the brown colour is completely uniform.

**Figure 12:**
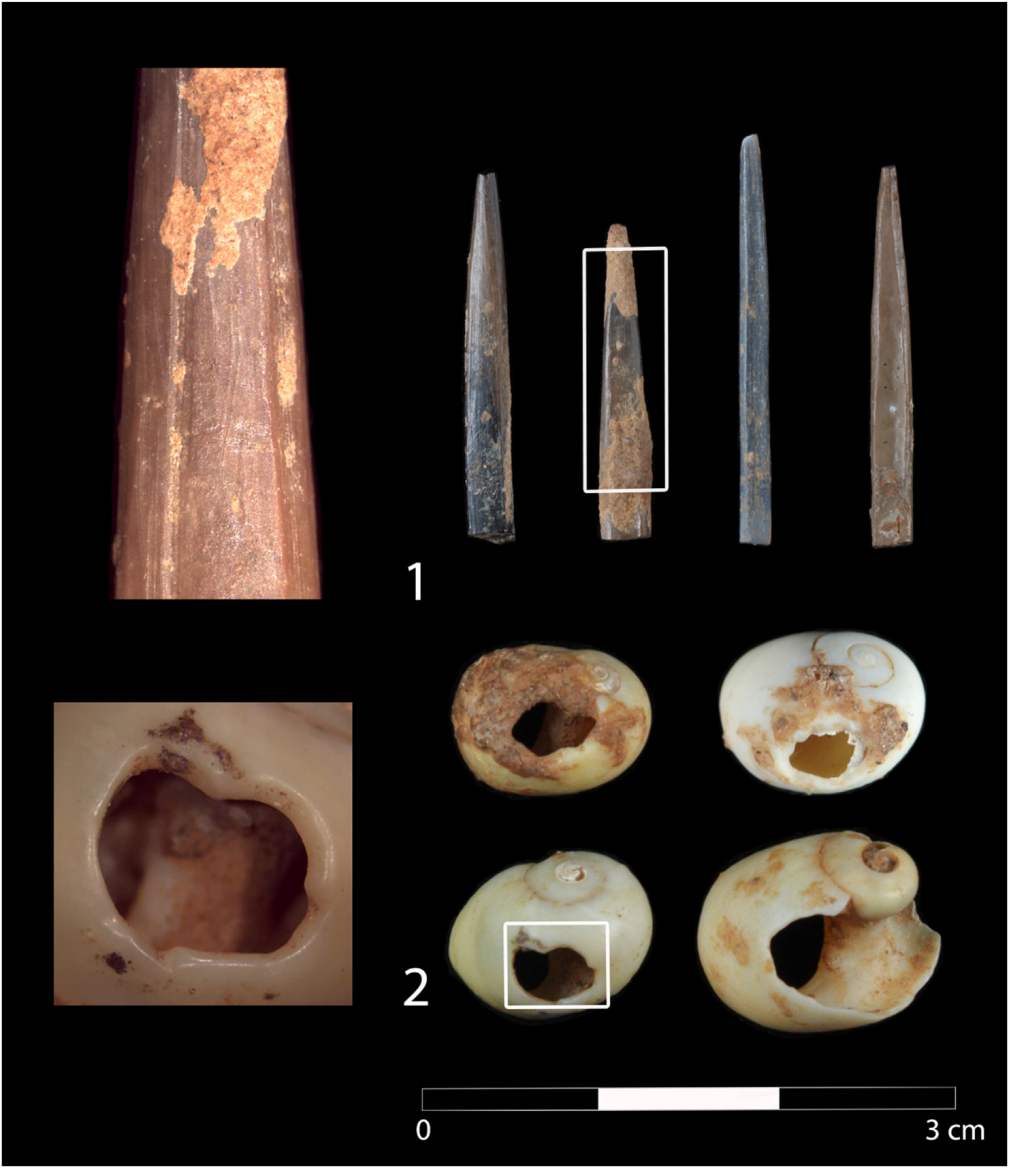
Bone points and objects of adornment from Phase (CV-shell-midden, CV-III and CV-IIIc) and Phase B/C (CV-IV) at Cueva Victoria.

#### 5.1.2 Pierced shells

The three shells of the marine mollusc *L. obtusata* were found in the Phase B (CV-III, CV-IIIc) and one in Phase B/C (CV-IV) (Figure 12. 2). One sheel of Tritia sp. (CV-Shell Midden, Phase B) is also perforated. These species were selected to make beads. All the shells display anthropic perforations in the area of the lip, on the side opposite to the aperture. Prior experimentation with shells of the same species has determined that the holes at *L. obtusata* were made by percussion from the outer surface of shells and were then enlarged with a pointed lithic implement. The two shells from CV-III and the shell from CV-IIIc display gloss around the entire perimeter of the hole and in the area of the shell mouth, whereas the one from CV-IV only has gloss in the part of the hole nearest to the edge of the mouth and in the region of the aperture. This gloss is thought to have been caused by the suspension of the shells during an indeterminate time. The perforation of *Tritia* sp. was made by pression, but it is broken.

### 5.2 Lithic assemblage

192 lithic remains have been documented (Table 4). This is a small assemblage but with very characteristic technological and typological traits. 156 of the remains are of flint (79.6%). The rest of the lithic assemblage is formed by objects in schist (9.3%), marble (4.1%), limestone (3.1%), quartzite (1.0%) and iron oxide, possibly siderite (1.0%). These raw materials can probably the rock strata of the Alpujarride and Malaguide complex. They appear in the form of pebbles, plaques and boulders which were brought to the cave to be used.

**Table 4:** Lithic assemblage from Cueva Victoria.

Phase	Level	Flint	Schist	Iron oxide	Marble	Limestone	Quartzite	Total
Phase A	CV-Superficial	9						*9*
Phase B	CV- Shell-midden	8	4		4	1	1	*18*
	CV-III	85	2	1		2	1	*91*
Phase B/C	CV-IV	12						*12*
Phase C	CV-V	39	6	1	4	3		*53*
	CV-V/VI	2	6					*8*
	CV- VII	1						*1*
	Total	**156**	**18**	**2**	**8**	**6**	**2**	**192**

27 remains in the flint assemblage do not provide any technological information as they are fragments of indeterminate objects, flakes or fragments of flakes small than 1cm in size, thermal fragments, *cassons* or burin spalls. Therefore, the study has analysed the other 129 artefacts. Seven flint types have been discriminated (Table 5) and the remains are in a good state of conservation although some pieces are affected by heat.

**Table 5:** Flint types identified in the sequence at Cueva Victoria.

TYPE	TRANSPARENCY	COLOUR	GRAIN	STRUCTURE	IDENTIFIABLE INCLUSIONS	CORTEX
CV1	opaque	greenish-grey	fine	heterogeneous	none	eroded
CV2	opaque	beige	coarse	heterogeneous	none	eroded
CV3	translucent	white	fine	homogeneous	none	?
CV4	opaque	white	medium	heterogeneous	quartz	?
CV5	translucent	brown	fine	homogeneous	none	eroded
CV6	from opaque to translucent	beige-reddish and yellow	fine	heterogeneous	none	?
CV7	opaque	grey-black	fine	heterogeneous	foraminifera	?

CV1 is the most abundant type in the assemblage, as it has been identified in the case of 97 objects. The second most common type is CV5, with 20 objects. The other types are represented by a maximum number of three objects each.

The assemblage consists of cores (n = 8), knapping maintenance pieces (n = 8), knapped blanks (n = 79), and retouched artefacts (n = 34). Two of the cores are tested nodules. Another one displays severe thermal alteration, and therefore only six cores provide information about the reduction strategy. Two of them were used to obtain blades, which were extracted from the wide face of the core. The others were used to obtain flakes. Centripetal reduction is observed in one of them, while another obtained small flakes by a semi-enveloping reduction (of the carinated endscraper type), and the others were worked on their wide face also to obtain small flakes. Only eight objects can be related to actions for core maintenance: four crests, three to repair the knapping surface and one to repair the percussion platform. Most of the blanks are flakes (n = 54), including some that tend towards a laminar form, while the other blanks are blades (n = 25). Most of the latter were found in CV-III (n = 16), as well as in CV-V (n = 7), in CV-IV (n = 1) and in the shell-midden level (n = 1). Most of them are fragmented (n = 18) and therefore do not preserve their original length. However, in some cases it can be estimated that they were of considerable size (n = 13, >10 mm in width). In those cases, in which it can be perceived, the blade blanks were appreciably regular and straight in their shape.

**Figure 13:**
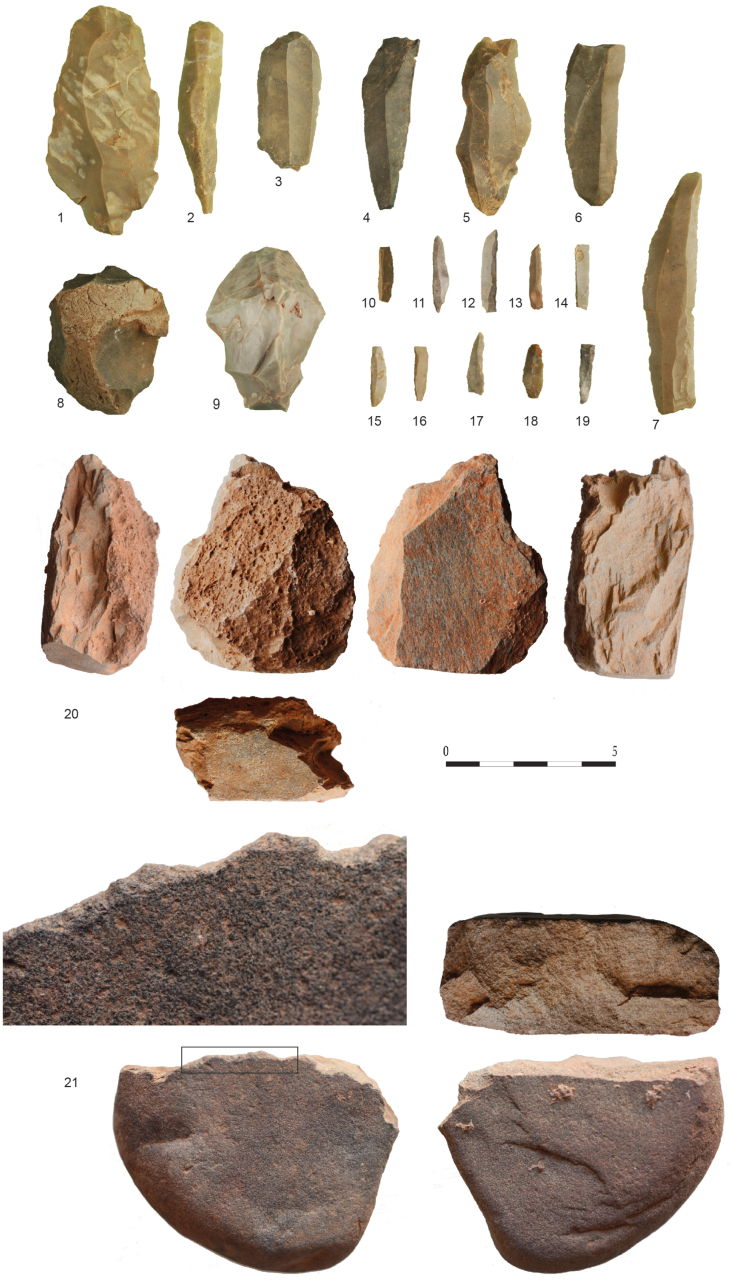
Lithic assemblage from Cueva Victoria. 1-3: end-scrapers; 4-6: burins; 7-8: cores; 9-18: armatures (backed bladelets); 19: blade; 20: rabot on schist; 21: knapped limestone pebble knapped with an edge rounded by use.

34 retouched tools have been studied (Table 6; Figure 13: 1-19) wich represent ca 21,8% of the total number of flint artefacts. Although the most numerous implements are armatures (n = 22), no particular elements or pointed objects can be distinguished as most of them are broken (Figure 13: 9-18). The three whole pieces are backed bladelets. The rest of the retouched assemblage consists of four endscrapers (Figure 12: 1-3), three burins, three notches-denticulated pieces and other two with continuous retouch. A few pieces fabricated in jasper (n = 3) have been detected in addition to the flint.

**Table 6:** Retouched tool groups from Cueva Victoria.

Phase	Level	Armatures	End scraper	Burins	Notches - Denticulates	Side scraper
Phase A	Superficial	2	0	0	0	1
Phase B	CV-Shell-midden	3	0	0	0	0
	CV-III	12	4	2	1	0
Phase B/C	CV-IV	0	0	0	0	0
Phase C	CV-V	5	0	1	2	1
	CV V-VI contact	0	0	0	0	0
	CV- VII	0	0	0	0	0
	TOTAL	**22**	**4**	**3**	**3**	**2**

In three cases, macrolithic tools shows clear evidence of their knapping and use: a chipped block of schist with alternating edges that create a *rabot*; a pebble, possibly of limestone/quartzite with evidence of chipping and edges rounded by use, also with disperse remains of ochre (Figure 12: 20-21); and a marble pebble fragment equally with ochre residues.

## 6 Palaeolithic rock art

The walls of Cueva Victoria are decorated with rock paintings attributed to the Upper Palaeolithic (Espejo and Cantalejo 1996; Cantalejo et al., 2007). The motifs are distributed in panels on eight different walls in the cave and represent two zoomorphs (a bovid and a bird), an anthropomorph, three panels with lines and stains made with fingers, another panel with dots also made with finger-tips, and the print of a left hand, together with a curved sign (Cantalejo et al., 2006). This small but interesting ensemble in Cueva Victoria was produced, in seven out of the eight panels, using finger-tips as a way to apply the red mineral pigment to the wall. The only case in which the use of a brush can be appreciated (a bird's feather has also been proposed) is precisely the depiction of a bird, which was identified as a cormorant. From the stylistic point of view, some of these representations are attributable to the time the cave was occupied in the late Pleistocene. Additionally, faded remnants of red paint can be observed in several parts of the cave but these could not be classified as graphic motifs when making the inventory of the ensemble.

The walls of Cueva Victoria were decorated again some millennia later, in the Neolithic. In this period schematic human figures in yellow and some weapons or tools associated with the anthropomorphs have been documented (Espejo and Cantalejo 1996; Márquez and Sanchidrián 2006; Cantalejo et al., 2007).

## 7 Discussion

### 7.1 Late Pleistocene chronology in the region

24 radiocarbon dates with a chronology between *ca.* 17.0 and 12.0 ka cal BP come from sites with very different information. The archaeological context with which they are associated is practically unknown in some cases (Zafarraya and Gorham's Cave) or discordant with the result of the date (Humo 6). This reduces the possibilities for an assessment to the samples from Nerja (Mina and Vestíbulo Chambers), Hoyo de la Mina and Cueva Victoria. Nonetheless, the number of dates and sites indicates a clear increase in human occupations during GI 1 in comparison with GS 2 and GS 3. With the present data, mainly from Nerja (Mina and Vestíbulo), the Magdalenian technocomplex in southern Iberia can be divided into the following phases (Aura, 1995; Aura et al., 2021).

- Magdalenian without harpoons or ‘A’, currently not known at coastal sites. Its estimated chronology is *ca.* 18.5–16.5 ka cal BP.
- Magdalenian with harpoons or ‘B’, dated between 14.8 and 13.5 ka cal BP. However, further north, the first harpoons in Valencia are dated in 16.5 ka cal BP (Cacho et al., 2001; Villaverde et al., 2012).
- Final Magdalenian without barbed points or ‘C’, with dates between 13.2 and 12.6 ka cal BP.
- Epipalaeolithic/Epimagdalenian, between 12.5 and 11.5 ka cal BP. The characteristics of the lithic and osseous assemblages display close similarities with the Magdalenian techno-economic traits.

The dates obtained for Phases B and C in Cueva Victoria can be related to the regional Magdalenian with barbed points. The date for CV-shell midden (*ca*. 13.8–13.6 ka cal BP) is on the boundary between the Upper and Final Magdalenian, and coincides with GS 1 (Aura et al., 2011). After 13.0-12-5 ka cal BP, the number of dates and known sites decreases, and this might be connected with taphonomic processes that have affected the cave deposits.

### 7.2 Palaeogeographic morphology and reconstruction of the region

The continental shelf near Cueva Victoria is relatively narrow, with a width of 8.5 km. Three segments have been differentiated: a) The inner-middle segment is gently sloping with mean gradients of 2°, and is characterized by the development of infralittoral prism-type sedimentary bodies and prodeltaic bodies associated with the mouths of the main rivers in the region; b) The middle-outer segment presents average slopes of around 0.5° together with the development of broad abrasion surfaces with rough morphologies, ridges, submarine terraces and depressions; and c) The outer segment and shelf-edge zone consists of a slope break located at approximately 110 m mean depth, with variations between 100 and 150 m, and is characterised by the development of prograding bodies that have been interpreted as marginal deltas or shelf-edge wedges.

Since the Last Glacial Maximum in the late Pleistocene, when sea level was located -120 m, there has been a steady rise until the present, with several stillstands and very short relative sea-level falls. The high-resolution relative sea level curve calibrated from the SPECMAP radiometric time scale by Thompson and Goldstein (2006) establishes that in the time interval when human occupation has been determined at Cueva Victoria, between 13.6 and 15.1 ka cal BP, the shoreline was located approximately 73 m below present-day sea level. The coastline location in this time interval is corroborated in this sector by the presence of submarine terrace levels with prograding sedimentary bodies according to the palaeogeographic reconstruction, in the time when the cave was occupied, the coastline would be located between 3 and 8 km from the present coast to offshore, and about 6 km from Cueva Victoria (Figure 7).

### 7.3 Palaeoenvironment

The meagre anthracological record does not allow any palaeoenvironmental reconstruction. Only one conifer fragment (possibly *Pinus* tp. nigra-sylvestris), two of *Quercus* and two of Fabaceae have been identified. However, the presence of these taxa can be discussed in comparison with other records in the region. These taxa are common in most anthracological sequences for the Late Glacial Period in the south-east of the Iberian Peninsula (Badal, 1990, 2006; Badal and Carrión, 2001; Badal and Martínez Varea, 2017). Several pine species (*Pinus nigra-sylvestris, Pinus pinea*, and with doubts *Pinus halepensis*) were detected in the anthracological ensemble from the Magdalenian levels in Cueva de Nerja (Mina and Vestíbulo Chambers). *Quercus* sp. is present in all the levels attributed to the GI 1, indicating its local presence, even though the vegetation was dominated by Fabaceae and other shrubs (Badal, 1990; Aura et al., 2002).

A point of reference for vegetation and palaeoenvironmental change in the area is Padul peat bog (Granada), where the regional expansion of different *Quercus* species has been documented from 18.0 ka cal BP onwards, together with a reduction in the pine curves (Camuera et al., 2019).

The terrestrial mammals in Cueva Victoria do not allow an interpretation in palaeoenvironmental terms. In the case of small vertebrates, their scarce presence in the deposit is noteworthy. The explanation could be found in the origin of the accumulation, since all the sediment recovered was water screened with 0.5 mm mesh sieve. Small vertebrate skeletons may accumulate by accidental deaths or by predation and deposition by owls or diurnal birds of prey, which display clear preferences regarding the characteristics of their nesting places. In the case of the different levels at Cueva Victoria, the possible absence of birds of prey (the few remains recovered could not be determined because they are covered with carbonate precipitation), and consequently the low number of small vertebrate fossil remains, could be explained by an increase in human activity in the surroundings, as has already been identified at El Cierro and Peñalarga caves (Álvarez-Fernández et al., 2020; Rofes et al., 2013). Neither can palaeoenvironmental interpretations be established from large mammals. Indeed, apart from aurochs and horse, the rest of the mammals documented in Cueva Victoria (ibex, red deer, wild boar, lynx, fox and rabbit) still live in the region. The same is true of the continental gastropods.

Some marine species are palaeoenvironmental change markers, demonstrating that the effect of the end of the Pleistocene was greater in the marine environment than on land. The gastropod *L. obtusata*, a species that is no longer present on Mediterranean coasts, is an indicator of more rigorous climate conditions on the coast of Malaga in the late Upper Palaeolithic than today (Jordá Pardo et al., 2011), when the maximum sea surface temperature would oscillate between 12 °C and 14 °C (Cacho et al., 2001), because of the entry of cold Atlantic water into the Mediterranean (Aura et al., 2002, 2016; Jordá Pardo et al., 2016). The presence of the whale balanoid *Tubicinella major* would form part of the same dynamic (Álvarez-Fernández et al., 2014).

### 7.4 Subsistence

The faunal remains found in Cueva Victoria show that a wide range of terrestrial and marine resources were used. A total of 5,960 remains have been classified. They are concentrated in Phase B, where they represent 65.8% of the total; while over 5,000 remains (ca. 89%) are of marine origin.

The assemblage of terrestrial remains includes different mammal and mollusc species. In Cueva Victoria, the number of terrestrial vertebrates that have been identified is small, but includes all those found in the regional Palaeolithic (Pérez-Ripoll and Martínez-Valle, 2001; Aura et al., 2002b). They are concentrated in Phase B, which accounts for >70%, rather than Phases A and C. The remains of rabbit come to over 90% of the NR, which concords with the situation at other sites (Pérez Ripoll, 2004). Phase B, with 73.2% of the identified remains in the sequence, has also yielded ibex, red deer, wild boar, aurochs and horse, although these are less abundant. This range of species, together with the presence of dolphin, resembles the results obtained in Nerja-Vestíbulo for the Magdalenian and Epipalaeolithic levels (Aura et al., 2002). However, a significant feature of the rabbit remains in Cueva Victoria is the low percentage of adult individuals and the frequency of sub-adults, juveniles and very young. This age profile does not match the data from other Upper Palaeolithic and Epipalaeolithic sites both in Andalusia (e. g. Pérez-Ripoll et al., 2011; Aura et al., 2009) and in the rest of Mediterranean Iberia (e. g. Pérez-Ripoll, 2004; Allué et al., 2010; Pérez-Ripoll and Martínez-Valle, 1995; Gordón, 2017; Martínez-Polanco et al., 2017; Nadihuska et al., 2018; Lloveras et al., 2016; Morales-Pérez, 2015) and in Portugal (Lloveras et al., 2011). At those sites where human action is the sole or main factor, adult rabbits predominate, while sub-adults are scarce and juveniles and very young are very rare. Consequently, it might be supposed that the abundance of sub-adult and juvenile individuals is related to the activity of carnivores and birds.

The diversity of herbivore species and small carnivores compared with the number of remains (NR) indicates that Cueva Victoria was not a specialised hunting site. However, the contribution to nutrition of the larger mammals, including the marine species, would have been greater than that of the smaller prey, like rabbits, and the marine molluscs, fish and crustaceans.

Few medium and large mammal remains were found. The species are represented by a very low NR and only *Capra pyrenaica*, *Cervus elaphus* and *Sus* sp. are present in different levels. These results coincide with the hunting identified in the region during the Late Palaeolithic (Aura et al., 2002). The samples do not allow an analysis of possible differences in the prey brought to the site related to the size of each species.

Similarly, the remains do not provide information about the seasonality of the occupations.

The presence of small carnivores, birds and the age of lagomorphs in the sample suggest that the cave was used for short occupations in which accumulations of their carcasses have not been observed.

The terrestrial molluscs would be collected by hand, probably in the vicinity of the cave.

In the case of marine molluscs, the two most abundant taxa, *Ruditapes decussatus* and *Solen* sp., would be collected from the mid-shore to the low shore zones. Both live burrowed in sand, but the former is also found in mud, gravel, or clay. They would have been foraged directly by hand, digging in the substrate, or by using digging tools, and using containers made with plant fibres and animal skins. The exploitation of the crabs was probably opportunistic. They were captured while the human groups were foraging bivalves on the shore.

Among the identified species of fish, the presence of sparids stands out. They would probably be caught with fishing rods in shallow coastal areas (posidonia meadows and on rocky substrates).

Shellfish and fish are predictable resources available throughout the year.

While shellfish-gathering and fishing were probably carried out individually, the possible acquisition of whale meat is likely to have been carried out in groups. The stranding of large cetaceans could well be an event in which different groups congregated to acquire their meat, skin and fat by means of lithic tools. The transport of this resource was probably carried out by several members of a community who would be in charge of transporting it to their settlement, where it would be processed.

The provision of animal resources would imply excellent knowledge of the ethology of the different species and, in the case of those of marine origin, the control of the tides.

These results suggest that Cueva Victoria was not a stable settlement, but rather a place that was occupied temporarily, with sufficient gaps for small carnivores and birds of prey to follow their natural biological behaviour in the cave or its surroundings.

The terrestrial gastropods of the species *Iberus alonensis* and *Iberus marmoratus*, found in the three phases in the cave (Phase B: 74.4%), were gathered in the proximity of the cave, where they were taken and consumed as food. This hypothesis is based on the selection of large species, the presence of adult individuals, the direct association of their shells with other remains taken to the cave to be consumed, and finds of thermally affected shells. The use of terrestrial molluscs as food is quite well known in the late Upper Palaeolithic in the south-east of the Iberian Peninsula. They are particularly abundant in Mina Chamber in Cueva de Nerja (Aparicio et al., 2015). This use was prolonged in time, as they have also been found in early Holocene levels at different sites in Mediterranean Spain (Fernández-López de Pablo et al., 2011; Balcázar-Campos et al., 2021).

The marine archaeozoological remains, the most abundant in the Cueva Victoria sequence, include molluscs, crustaceans, fish and a mammal.

The marine mollusc shells, found in all three phases, are the most numerous remains and about 65% of the total come from Phase B. Most of the species possess alimentary value (>99.5% of the NR and >97.5% of the MNI). Gathering concentrated on bivalves in sandy and muddy substrates in the mid-shore or low-shore zones of beaches near the cave, especially on clams (>97% of the NR; >95% of the MNI) and to a much lesser extent razor shells and cockles. The burnt shells found in the three phases are not the consequence of their preparation for consumption (opening the valves of the shells by heat) but the result of hearths located in the places where the shells had been left after the consumption of the flesh. The biometric analysis of the shells indicates a preference for adult individuals. Despite smaller sizes in the recent phases (A and B) compared with the older Phase C, this decrease is probably not due to over-exploitation of the species, as the difference in size is ≤ 2mm.

Shell-fishing is well documented at Upper Magdalenian sites on the Alboran Sea (Aura et al., 2016). The caves of Hoyo de la Mina and El Tesoro are close to Cueva Victoria. In the former, the abundant remains of edible invertebrates include clams (*R. decussatus*) as well as cockles (*Cerastoderma* sp.) and oysters (*Ostrea edule*), whereas the other species (among them the *Solen*, *Murex* and *Haliotis* genera) are much scarcer (Ferrer et al., 2006; Such, 1920). *R. decussatus, Cerastoderma* sp. *and Solen* sp. shells are cited for the latter cave (Aura et al., 2013). To the west of Cueva Victoria, 332 remains of marine shells, many of them affected by fire, were found in Rock-shelter 6 in the Humo Complex (Malaga). Among the thirty species classified, *P. vulgata* was a gastropod collected for its alimentary value (Ramos et al., 2006). However, Cueva de Nerja (Maro, Nerja) to the east of Cueva Victoria, has undoubtedly provided most information about shell-fishing in the area (Jordá Pardo, 1981, 1982, 1986; Jordá Pardo et al., 2016). In the Magdalenian levels in the Vestibule of the cave (NV 7, NV 6 and NV 5), bivalves gathered in sandy or muddy substrates are present. *R. decussatus* and *Cerastoderma* sp. are represented by 10% of the NR; *Solen* sp. is not present. In contrast, in the Upper Magdalenian levels of Mina Chamber (NM 16 to NM 14), molluscs gathered in sandy substrates predominate among the edible species (*ca*. 70% of the NR). Of these, clams make up >85% and cockles 12.5%.

In the rest of Mediterranean Iberia, molluscs gathered for food are much rarer in Upper Magdalenian deposits. One example is in Cueva del Caballo (Cartagena) for which clams, limpets, mussels, etc. have been cited (Martínez-Andreu, 1989; Román et al., 2020). At least 60% of the NR belong to species that potentially might have been collected for food.

In addition to the molluscs, other marine taxa documented in Cueva Victoria include remains of crustaceans: eight pincers of the *Carcinus* crab genus (in Phases B and C) and a plate of the whale balanoid *Tubicinella major*, in Phase A. The finds of pincers (the toughest part of the skeleton) of adult crabs might suggest the selection of the largest individuals. The fact that some of them are burnt should not be associated with their processing, as noted above for the clams. The relative scarcity of the remains may be related to fortuitous foraging during shell-fishing. Crabs are relatively common at Mediterranean Upper Magdalenian sites. They have been documented in Cueva de Nerja (Mina Chambers), where they are currently being studied. The crab *Eriphia verrucosa* and the balanus *Chthamalus stellatus* have been cited at Cueva del Caballo (Román et al., 2020), while indeterminate crustaceans have been described at Hoyo de la Mina (Such, 1920) and Volcán del Faro in Valencia (Davidson, 1973).

The single whale balanus plate documented in Cueva Victoria belongs to the species *Tubicinella major*, and indicates that the human groups scavenged a beached southern right whale (*Eubalaena australis*), to which this balanus is endemic, and a piece of meat with skin and fat to which the balanus was adhered was taken to the cave to be cooked. This crustacean only comes loose when the flesh of the whales is processed by fire (Jerardino and Parkington, 1993; Kandel and Conard, 2003), which explains why this plate in Cueva Victoria is affected by fire and fragmented. The presence of *Tubicinella major* had only been identified previously in Upper Magdalenian levels in Mina Chamber in Cueva de Nerja, where over a hundred plates and fragments of plates belonging to a minimum of 13 individuals were identified (Álvarez-Fernández et al., 2014).

Fish remains are relatively scarce (n = 57), and while present in the three phases, 87.5% were found in Phase B. They are all marine taxa and some of the remains are fragmented and thermally altered. It is possible that the profound carbonation of the bioarchaeological remains has influenced the recovery of smaller items. The importance of the sparids indicates fishing on rocky substrates next to the shore. Sparids are the most abundant sea fish in the Upper Magdalenian sequences in Cueva de Nerja both in the Vestibule (NV7-NV5) and in Mina Chamber (NM 16–14), where they amount to nearly 50% of the NR of marine fish (Aura et al., 2002).

Bird remains are equally rare and found only in Phase B. Owing to their poor state of conservation, the agent that brought them to the cave cannot be determined. However the use of birds (as food, for their feathers, etc.) has been documented in Nerja in the Magdalenian levels of both Mina Chamber (Eastham, 1986) and in the Vestibule (Morales-Pérez et al., 2020).

Finally, the only evidence of a marine mammal in Cueva Victoria is a vertebra fragment from a short-beaked common dolphin, found in CV-shell midden and therefore attributed to Phase B. This species has been recorded in the Upper Magdalenian levels in Vestíbulo and Mina Chambers in Cueva de Nerja: 18 remains (3 mandibles, 13 teeth and 2 vertebrae) in NM 16, a mandible fragment in NM 14, and 12 remains in NV 7–4 (Aura et al., 2009, 2016).

The fauna in Cueva Victoria enlarges our knowledge of the economy of the Magdalenian groups that occupied the sites on the shore of the Alboran Sea. According to the data for Cueva de Nerja, this was based on the exploitation of marine resources (Aura et al., 2002), as observed in the early 20^th^ century in Hoyo de la Mina (Such, 1920) and described more recently in La Araña/Humo Complex (Ferrer et al., 2006; Ramos et al., 2006). At the other El Cantal/La Cala sites (Cueva del Tesoro and Cueva del Higuerón) accumulations of marine remains have been cited but no updated studies are available. Two factors help to understand the formation and conservation of these shell-middens from the Pleistocene-Holocene transition. As described above, the bathymetry and morphology of the continental margin has allowed the conservation of the sites. The entry of Atlantic water rich in nutrients has also been related to this concentration of sites on the southern Iberia coast (Aura et al., 2016).

Cueva Victoria was a little further from the coast at the time of the occupations (±6 km) and taphonomic data from the study of the terrestrial fauna indicate short human occupations, based on the age of the rabbits and the presence of carnivores. This differs from the situation at Cueva de Nerja, about 3.5km from the shore in GI 1 (Jordá Pardo et al., 2011). This is a site with thick archaeological deposits, high occupation densities and evidence of continuity in lithic and osseous industry. They are sites that display significant differences but which provide complementary data about regional mobility and subsistence.

The exploitation of beached marine mammals at both sites (especially cetaceans, but also dolphins and seals) exemplifies the marine orientation of these groups and suggests hypotheses about the influence of the cyclical-seasonal nature of these beachings on mobility and the location of the occupations. They would undoubtedly have been an attraction for the concentration of groups dispersed in inland areas.

At present, southern right whales live in the southern Atlantic Ocean and migrate annually towards the Equator in winter. The presence of plaques of the balanus *Tubicinella major*, endemic to this whale, in Nerja-Mina, was explained by the entry into the western Mediterranean of an individual that crossed the Equator at some point in the GI 1 (Álvarez-Fernández et al., 2014). Now, the find of a *Tubicinella major* plate in Cueva Victoria suggests two hypotheses. First, it might have come from a different southern right whale from the one that beached near Cueva de Nerja, and therefore the entry of these whales into the Mediterranean during the GI 1 was not so exceptional. Second, the balanus remains in the two caves came from the same whale that reached the coast near Nerja and beached there. It would have been seen by hunter-gatherers occupying the sites along the coast (at least in Cueva Victoria and Cueva de Nerja) who met to make use of and share out its meat and fat (to which the *Tubicinella major* specimens were attached). They took the resources to their respective caves where they cooked them. The radiocarbon dates from the levels in the two caves with the balanus remains supports the first hypothesis, although they were obtained from associated remains; pine nuts in Nerja (NM 16) and shells in Cueva Victoria (CV-surface), not directly from the balanus remains.

### 7.5 Technology: bone and lithic gears

Evidence of industry in bone and shells has only been documented in Phase B at Cueva Victoria.

The four bone objects that have been documented come from the medial and end parts of the artefacts, but it is not possible to determine if the latter are the points or the bases of them. None of them conserve a perforation and therefore they cannot be classified as needles with a pierced head. Consequently, they may be regarded as fine, and possibly double, points. In the area of their ends they display polish which is probably interpreted as use-wear. The association of this type of artefact with occupations oriented towards the exploitation of the marine environment, and deposits formed by thousands of remains of invertebrates, fish, birds and mammals, has led to their identification as straight fish hooks or gorges (Aura and Pérez, 1998; Barandiarán, 2002; Averbouh, 2003). Similar artefacts in their technique, shape and size have been found in Mediterranean upper Magdalenian levels with barbed points and Epipalaeolithic/Epimagdalenian levels in Cueva de Nerja (Vestíbulo and Mina Chambers) and Hoyo de la Mina (Aura et al., 2013). These levels coincide in their radiocarbon chronology with the age of Phase B in Cueva Victoria.

The four specimens of the sea snail *L. obtusata*, and one *Tritia* sp., both of them small species with no alimentary value, had been made into beads and then used, as indicated by the gloss around their perforations. Shells of this species were often used as objects of adornment at Magdalenian sites in the Iberian Peninsula; for example in Malaga at Rock-shelter 6 in the Humo Complex (Ramos et al., 2006) and in the Vestibule at Cueva de Nerja (Jordá Pardo et al., 2010), in other regions (e.g., Cueva de El Caballo, in Murcia: Martínez, 2015), and even at sites some distance from the sea, e.g., Montlleó in the lower Magdalenian (García-Argudo, 2018) and Chaves, in the upper Magdalenian (Álvarez-Fernández, 2006), both located on tributaries to the River Ebro.

The lithic assemblage at Cueva Victoria is small but displays very characteristic traits that are easily compared with other regional assemblages (Aura et al., 2011). Local raw materials were used. The volumes and the weathered cortex indicate that flint cobbles might have been collected on beaches or at the mouths of the valleys, like the Totalán valley. Rocks with a coarser grain (slate, quartzite and limestone) were also used to make more expeditious implements. These macrolithic artefacts display evidence of use very similar to those described for Cueva de Nerja (Aura and Jardón, 2006).

The knapped lithic assemblage was made on medium-high quality flint. It was used to obtain blade or bladelets with which the main groups of tools were made (Figure 12). However, a large number of flakes and cores used to obtain them have also been found. These may have been produced to maintenance the laminar debitage or represent the final use of blade-bladelet cores.

The debitage system and the tools found at Cueva Victoria, a little over thirty pieces, are very similar to those documented in the upper Magdalenian and Epipalaeolithic levels at Cueva de Nerja (Aura, 1986; Aura et al., 2013). The typological indices reflect some general patterns but with significant variability. In general, burins appear in similar numbers or are more numerous than the endscrapers. The assemblages are dominated by armatures made from bladelets, with the other groups (truncated pieces, retouched pieces, etc.) appearing in different proportions. They are standardised productions that used good quality flint, reduced with hard hammerstones (Aura and Jardón, 2006; Vadillo et al., 2019). The high percentage of retouched artefacts in the total flint lithic assemblage (21.79%) may be related with an assemblage primarily deposited during ephemeral occupations (Barton et al., 2018).

In sum, the production methods for the lithic and osseous industry, and the frequencies of the typological groups, mean that the materials from Cueva Victoria are coherent with the regional Magdalenian. Radiocarbon dates practically coincide with those obtained at Cueva de Nerja, where the characteristics of the Upper Magdalenian with harpoons and the final Magdalenian have been defined (Aura et al., 2013).

### 7.6 Artistic representations

A part of the parietal ensemble in Cueva Victoria can be attributed to the Palaeolithic from both the thematic point of view, with a predominance of abstract over figurative depictions, and their technique. These types of representations have been documented in other areas in south-east Iberia, as they are currently known at over forty sites in the provinces of Cádiz (28), Málaga (10), Granada (1), Jaén (1) and Almería (1), as well as in Gibraltar (1). Two clear clusters can be differentiated in the mountains of Málaga (in the dark zone of caves) and near the Strait of Gibraltar (in open-air rock-shelters), and these total 38 sites. Good examples are the sites of Navarro IV (Sanchidrián, 1982) and Calamorro (Fortea and Giménez, 1973) in the Province of Málaga, Estrellas/Tajo de las Abejeras (Collado et al., 2019; Fernández et al., 2019) and Palomas in Cádiz (Breuil and Burkitt, 1929; Collado et al., 2020), and Malalmuerzo (Cantalejo, 1983; Cabello et al., 2019) in Granada. Compared with these types of rock art sites with small Palaeolithic parietal ensembles, large caves like La Pileta, Nerja and Ardales demonstrate the continuity in graphic aggregations that have formed ensembles with thousands of Palaeolithic motifs (Cantalejo and Espejo, 2014).

## 8 Conclusions

The study of Professor Fortea's 1972 excavations in Cueva Victoria (Sala de las Conchas) has obtained significant chrono-stratigraphic and techno-economic information about the late Palaeolithic in the south of the Iberian Peninsula.

From the chrono-stratigraphic point of view, the levels observed in the cave were sealed by successive episodes of the formation of flowstone, in which the radiocarbon dates are coherent with the stratigraphic position of the dated samples (Phases A, B and C). There is no evidence of artefact (e.g. pottery, polished tools, etc.) that indicate Holocene occupations at Sala de las Conchas. This dynamic of sedimentation and dates coincides with the studies of the fauna and lithic and osseous industries, as they indicate the formation of palimpsests generated by reiterated human occupations, as well as times when the cave was unoccupied and it was used by carnivores and birds of prey.

An argument supporting this interpretation of the occupations at Cueva Victoria is the great diversity of terrestrial mammal species within a small NR. Together with the ages at death of a small prey (*Oryctolagus cuniculus*), this is indicative of alternating human and carnivore uses of the cave. These short occupations, as also suggested by the high frequency of retouched tools, are not incompatible with the increase in the number of sites observed at regional level in the GI 1.

The different palaeoenvironmental proxies obtained from our research and from the investigations carried out at other sites in the SE of the Iberian Peninsula indicate temperate conditions in this territory during GI 1. Here the Mediterranean landscape predominated. Despite the fact that the archaeobotanical information obtained in Cueva Victoria is scarce, from a regional point of view it can be extrapolated with information of this type from the neighbouring Nerja Cave and other sites located further north (Aura et al., 2005; Badal and Martínez Varea, 2017), where thermophile taxa predominate. In the case of the archaeozoological evidence recovered in Cueva Victoria (mammals, terrestrial gastropods), the information is much more significant. Together with data from other deposits, it confirms those temperate conditions that existed during the late Pleistocene. However, this contrasts with the existence of cold-loving taxa in the marine environment, as demonstrated by marine invertebrate remains recovered at Cueva Victoria and at Nerja.

The identification of marine species tolerant of colder water (cf. *Littorina obtusata*) or directly Atlantic species (cf. *Tubicinella major*) shows that palaeoenvironmental changes took place at different rates at marine and continental scales (Aura et al., 2002). The coastline reconstruction differs somewhat from the situation known for Cueva de Nerja Cave but this can be explained by variations in the morphology of the continental margin near Cueva Victoria, with more beaches compared with the cliffs and small coves described for Nerja. This explains the gathering of molluscs from sandy substrates in GI 1, contrasting with the bivalves from a rocky substrate documented at Nerja during the GS 1 occupations (Jordá Pardo et al., 2016).

The faunal studies indicate short human occupations that combined the exploitation of terrestrial and marine resources. The biometric study of the bivalves (cf. *Ruditapes decussatus*) shows that there is no statistical evidence of their over-exploitation at Cueva Victoria. Fishing was another subsistence activity, aimed at coastal species, while crustaceans and echinoderms would also have been collected on beaches. This subsistence pattern differentiates a cluster of sites on the Andalusian coast of the Alborán Sea with singular techno-economic characteristics that have not been identified on the rest of the Spanish Mediterranean coast.

The lithic and osseous technology system identified at Cueva Victoria coincides with the regional Mediterranean Magdalenian techniques and possesses common elements with Nerja in the upper Magdalenian and Epipalaeolithic. Characteristics such as the use of local raw materials, reduction systems aimed at obtaining laminar-microlaminar blanks, the use of pebbles gathered on beaches and the manufacture of fine bone points are some of the clearest coincidences. These technical traits are shared with the other regional El Cantal/La Cala sites.

This regional cluster is also characterised by artistic engravings and paintings widely distributed at coastal and inland sites, even over 50km from the modern coastline. A small number of depictions of marine fauna (fish, birds and mammals) are known but their distribution is associated with the exploitation of marine resources in the deposits conserved on the shore of the Alborán Sea. These resources include the use of cetaceans, inferred from the skeletal remains of a dolphin and a balanus from a southern right whale.

This systematic use of marine resources might have influenced the mobility of groups along the coast. Unfortunately, no data is available for the inland sites, between 30 and 50km from the coastline, which could be used to assess the importance of marine resources at those interior occupations and their relationship with the coastal groups.

## 9 Materials and methods

### 9.1 Materials

All the studied materials were obtained by Professor Fortea Pérez in his excavation in Cueva Victoria in summer 1972 and since then they have remained on deposit in the Department of Prehistory and Archaeology at the University of Salamanca. The excavation logbooks in Professor Fortea's personal archive have also been consulted in order to carry out the present study.

### 9.2 Methods

#### 9.2.1 Geology, stratigraphy and archaeological sequence

The geological surroundings of the site have been determined with the Continuous Geological Map of Spain at 1:50,000 scale, of the Spanish Geological and Mining Institute and its cartographic viewer (http://info.igme.es/visorweb/). The stratigraphy in the cave has been identified in visits to the cave but no archaeological excavation has been carried out. The stratigraphic and archaeological sequence has therefore been reconstructed with the information in Professor Fortea's 1972 excavation logbook.

#### 9.2.2 Palaeogeography

The determination of the coastline in the established time interval of human occupation at the Cueva Victoria site was based on the SPECMAP-correlated northern hemisphere sea level variation curve over the last 30,000 years by Thompson and Goldstein (2006). Paleogeographic reconstruction and digital bathymetric model was carried out using data from the EMODnet Bathymetry portal (http://www.emodnet-bathymetry.eu).

#### 9.2.3 Radiocarbon

Three samples taken from the archaeological remains obtained in Professor Fortea's excavation have been analysed in the Oxford Radiocarbon Accelerator Unit (ORAU) Laboratory at the University of Oxford. According to the labels on the bags with the remains, the samples come from the superficial level (“shell-midden”), Level IIIc in Square D4 and Level Vc in Square A1. The results were subjected to a validity test (Mestres 1995, 2000, 2003, 2008) to verify that they fulfil the basic chemical-physical, analytical and archaeological requirements. In this way, all three samples correspond to organic material (marine bivalve shells of the species *Ruditapes decussatus*) which fulfils the chemical-physical requirements. They also comply with analytical demands, as the ORAU Laboratory is certified by the ISO-9001 Quality Management benchmark by the British Standards Institution for radiocarbon measurements, which is the highest quality standard that an analytical laboratory can reach in the United Kingdom. Regarding the precision of the dates, two have standard deviations of 55 years and the other of 45 years. Finally, in terms of the archaeological requirements, there is no reasonable doubt that that the three shells are associated with anthropic activity in the levels of the deposit and that they are all organisms with a short life, and therefore can be considered synchronic with that activity. Two of them are altered by heat owing to exposure to fire.

#### 9.2.4 Anthracology

The charcoal fragments were obtained when breaking up pieces of calcited shell-midden and processing the sediment from each level. The charcoal remains were manually fractured by hand along the three anatomical observation planes: transversal, tangential and radial sections and wood anatomical features compared with specialised literature on plant anatomy (Schweingruber, 1990; Vernet et al., 2001) and a reference collection of current charred wood. The analysis was carried out with a LEICA reflected light brightfield/darkfield optical microscope with different lenses ranging from 50x to 1000x magnification supported by SEM microscopy in some cases. Nomenclature follows the guidelines in Flora Europaea (Tutin et al., 1964).

#### 9.2.5 Archaeofauna

Identifications have been made with the reference collections held by the different hosting institutions of some of the authors of this paper. The study of micromammals and reptiles has followed Sese (2005). Hoyo et al. (1992–2010) has been followed for the birds. For large mammals, several osteological atlases have also been used (e. g. Pales and Garcia, 1981; Barone, 1976; Lavocat, 1966; Hillson, 1996; Schmid, 1972; and the reference collection in the Department of Prehistory, University of Valencia). Le Gall (1984) has been followed for the fish remains. For the determination of the invertebrate remains, different publications were considered: e. g., Moreno (1994) and Jordá Pardo et al. (2016) for the molluscs; Álvarez-Fernández et al. (2014) for the barnacle; Gruet and Laporte (1996), for the decapoda remains. Minimum Number of Individuals (MNI) was used for quantification although the Number of Remains (NR) has also been considered. Taphonomic analysis has observed anthropic modifications, thermal alterations and carnivore damage.

Information about the biotopes of the different taxa has been taken from specific studies for large mammals (Svenning et al., 2016; Granados et al., 2004; Pagel et al., 1991; Carranza, 2011), small mammals (Soriguer, 1983), fish (e.g. Doadrio 2002), birds (e. g. Hoyo et al., 1992–2010, terrestrial molluscs (e. g. Welter-Schultes, 2012), marine molluscs (e. g. Gofas et al., 2011a and b) and crustaceans (e. g. Zariquiey Álvarez, 1968; Southward, 2008).

The nomenclature of FAUNA EUROPAEA was followed for large mammals, birds and continental molluscs (Fauna Europaea version 2017.06, Zoological Museum Amsterdam/University of Amsterdam, http://www.faunaeur.org; retrieved 06-20-2020). In the case of small mammals, the work of Lavocat (1966) has been used, whereas the study of reptiles has followed Speybroek et al. (2010). WoRMS nomenclature was used for the marine invertebrates (WoRMS Editorial Board, 2019). Fish classification followed the Eschmeyer Catalog of Fishes (http://www.calademy.org/scientists/catalog-of-fishes-classification/; retrieved 06-20-2020).

Finally, biometric data were collected for well-preserved marine bivalve shells in order to establish criteria for an exploration of possible size changes in the species in the course of the sequence and to determine their possible causes. Statistical tests (Kolmogorov-Smirnov and ANOVA test) were applied for this purpose.

#### 9.2.6 Lithic industry

The chipped lithic assemblage has been studied technologically and typologically. First, the technological categories that define the stages in the operational chain have been identified (Perlès, 1991; Inizian et al., 1995; Pelegrin, 2000). Then, the detailed analysis of the cores has enabled an appreciation of the debitage objectives (flakes or blades) and the type of reduction, depending on the characteristics of the active face (Vadillo, 2018). A typological study of the retouched artefacts has also been performed (Sonneville-Bordes, 1960). The most abundant raw material, flint, has been characterised macroscopically based on such variables as transparency, colour, structure, presence of inclusions and type of cortex.

#### 9.2.7 Bone industry and personal ornaments

The study of the osseous assemblage has followed specific studies on bone points in the Mediterranean region (Aura and Pérez 1998; Villaverde et al., 2016). The study of the personal adornments has followed Álvarez-Fernández (2006) and Avezuela et al. (2011), which analyse in detail the ornaments made from marine gastropod shells.

## Declarations

### Author contribution statement

Esteban Álvarez-Fernández, J. Emili Aura Tortosa, Jesús F. Jordá Pardo, Pedro Cantalejo and Adolfo Maestro: Conceived and designed the experiments; Performed the experiments; Analyzed and interpreted the data; Wrote the paper.

Mª Teresa Aparicio, Yolanda Carrión, Mª José Fernández-Gómez, F. Javier Martín-Vallejo, Xabier Murelaga, Ismael Palomero-Jiménez, Manuel Pérez-Ripoll and Margarita Vadillo Conesa: Conceived and designed the experiments; Performed the experiments; Analyzed and interpreted the data.

Lidia Cabello-Ligero, María del Mar Espejo, Naroa García-Ibaibarriaga and Ricard Marlasca: Performed the experiments; Analyzed and interpreted the data.

### Funding statement

This work was supported by the University of Salamanca GIR PREHUSAL, the Ministry of Science and Innovation-Spanish Government (PaleontheMove-PID2020-114462GB-I00), the Universidad Nacional de Investigación a Distancia (Madrid) and Direcció General de Universitat, Investigació i Ciència of the Valencian Regional Government (Project Aico/2020/97).

### Data availability statement

Data included in article/supplementary material/referenced in article.

### Declaration of interests statement

The authors declare no conflict of interest.

### Additional information

No additional information is available for this paper.

## References

[bib1] Allué E., Ibáñez N., Saladié P. (2010). Small preys and plant exploitation by late Pleistocene hunter–gatherers. A case study from the Northeast of the Iberian Peninsula. Archaeol. Anthropol. Sci..

[bib2] Alonso B., Ercilla G., Baraza J., Maldonado A., Maldonado A. (1992). El Mar de Alborán y el Golfo de Cádiz: Conexiones Atlántico-Mediterráneo. III Congreso Geológico de España.

[bib3] Álvarez-Fernández E. (2006).

[bib4] Álvarez-Fernández E., Carriol R.P., Jordá Pardo J.F., Aura J.E., Avezuela B., Badal E., Carrión Y., García-Guinea J., Maestro A., Morales J.V., Pérez G., Pérez-Ripoll M., Rodrigo Ma.J., Scarff J.E., Villalba Ma.P., Wood R. (2014). Occurrence of whale barnacles in Nerja Cave (Málaga, southern Spain): indirect evidence of whale consumption by humans in the Upper Magdalenian. Quat. Int..

[bib5] Álvarez-Fernández E., Bécares J., Jordá Pardo J.F., Agirre-Uribesalgo A., Álvarez-Alonso D., Aparicio Ma T., Arias P., Barrera-Mellado I., Carral P., Carriol R.-P., Cubas M., Cueto M., Douka K., Elorza M., Fernández-Gómez, M J., Gabriel S., García-Ibaibarriaga N., Iriarte-Chiapusso, Ma J., Llave C., Maestro A., Martín-Jarque S., Portero R., Suárez-Bilbao A., Tarriño A., Uzquiano P. (2020). Palaeoenvironmental and chronological context of human occupations at el Cierro cave (northern Spain) during the transition from the late upper Pleistocene to the early Holocene. J. Archaeol. Sci. Rep..

[bib6] Aparicio M.T., Álvarez-Fernández E., Jordá Pardo J.F., Avezuela B., Aura J.E., En, Gutiérrez F., Cuenca D., González M. (2015). La Investigación Arqueomalacológica en la Península Ibérica: nuevas aportaciones.

[bib7] Archer W., Braun D.R., Harris J.W.K., McCoy J.T., Richmond B.G. (2014). EarlyPleistocene aquatic resource use in the Turkana Basin. J. Hum. Evol..

[bib8] Aura J.E., Jordá Pardo J.F. (1986). La Prehistoria de la cueva de Nerja (Málaga). Trabajos sobre la cueva de Nerja 1: 205-267. Patronato. de la Cueva de Nerja, Málaga.

[bib9] Aura J.E. (1995).

[bib10] Aura J.E., Jardón Giner P., Sanchidrián Torti J.L., Márquez Alcántara A.M., Fullola Pericot J.M. (2006). La cuenca mediterránea durante el Paleolítico superior 38000- 10000 años: 284-297.

[bib11] Aura J.E., Pérez C.I., Sanchidrián J.L., Simón Ma D., coord (1998). Las culturas del Pleistoceno superior en Andalucía.

[bib12] Aura J.E., Jordá J.F., Pérez M., Rodrigo M.J., Badal E., Guillem P. (2002). The far south: the pleistocene–holocene transition in Nerja cave (andalucía, Spain). Quat. Int..

[bib13] Aura J.E., Carrión Y., Estrelles E., Pérez Jordà G. (2005). Plant economy of hunter-gatherer groups at the end of the last Ice Age: plant macroremains from the cave of Santa Maira (Alacant, Spain) ca. 12000-9000 BP. Veg. Hist. Archaeobotany.

[bib14] Aura J.E., Jordá Pardo J.F., Morales J.V., Pérez Ripoll M., Villalba Currás M.P., Alcover J.A. (2009). Economic transitions *in finis terra*: the western mediterranean of Iberia, 15 – 7 ka BP. Before Farming.

[bib15] Aura J.E., Jordá Pardo J.F., Montes L., Utrilla P. (2011). Human responses to younger dryas in the Ebro valley and mediterranean watershed (eastern Spain). Quat. Int..

[bib16] Aura J.E., Jordá Pardo J.F., Pérez-Ripoll J., Badal E., Tiffagom M., Morales J.V., Avezuela B., Rasilla M., Javier Fortea Pérez F. (2013). Universitatis Ovetensis Magister. Estudios en homenaje.

[bib17] Aura J.E., Jordá Pardo J.F., Álvarez-Fernández E., Pérez Ripoll M., Avezuela B., Morales J.V., Rodrigo Ma.J., Marlasca R., Alcover J.A., Jardón P., Pérez Ma.J., Pardo S., Maestro A., Villalba Ma.P., Salazar-García D.C. (2016).

[bib18] Aura J.E., Vadillo M., Morales-Pérez J.V., En Roman D. (2020). Las facies microlaminares del final del Paleolítico en el Mediterráneo ibérico y el valle del Ebro 199-230, Monografies SERP.

[bib19] Aura J.E., Jordá Pardo J.F., Vadillo M. (2021).

[bib20] Averbouh A., Clottes J., Delporte H. (2003). La Grotte de la Vache (Ariège). Fouilles Romain Robert. I.- Les occupations du Magdalénien.

[bib21] Avezuela B., Martín I., Marín J.A., Muñoz F., Morgado A., Baena J., García D. (2011). La investigación experimental aplicada a la arqueología.

[bib22] Ayala Carcedo F.J., Rodríguez J.M., Val J. del, Durán J.J., Prieto C., Rubio J. (1986).

[bib23] Badal E. (1990).

[bib24] Badal E., Carrión J.S., Fernández S., Fuentes N., coords (2006). Paleoambientes Y Cambio Climático.

[bib25] Badal E., Carrión Y., Villaverde V. (2001). De Neandertales a Cromañones. El inicio del poblamiento humano en tierras valencianas.

[bib26] Badal E., Martínez Varea C.M. (2017). Different parts of the same plants. Charcoals and seeds from Cova de les Cendres (Alicante, Spain). Quat. Int..

[bib27] Bailey G.N. (2004). World prehistory from the margins: the role of coastlines in human evolution. J. Interdiscip. History Archaeol..

[bib28] Bailey G.N., Milner (2002). Coastal hinter-gatherers and social evolution: marginal or central?. Before Farming.

[bib29] Balcázar-Campos N., Aparicio M.T., Aura Tortosa J.E. (2021). Primeros datos sobre la malacofauna terrestre de Coves de Santa Maira (Castells de Castells, Alacant) durante la transición Pleistoceno-Holoceno (15 - 6 ka cal BP). Soc. d'Hist. Nat. Bal..

[bib30] Barandiarán Maestu I., Cava A., Barandiarán I. (2002). Cazadores-recolectores en el Pirineo navarro: el sitio de Aizpea entre 8000 y 6000 años antes de ahora.

[bib31] Bárcenas P. (2013).

[bib32] Bárcenas P., Fernández-Salas L.M., Macías J., Lobo F.J., Díaz del Río V. (2009). Estudio morfométrico comparativo entre las ondulaciones de los prodeltas de los ríos de Andalucía Oriental. Rev. Soc. Geol. Espana.

[bib33] Barone R. (1976).

[bib34] Barroso Ruíz C. (2003).

[bib35] Barton C.M., Aura J.E., Garcia-Puchol O., Riel-Salvatore J.G., Gauthier N., Conesa M., Pothier Bouchard G. (2018). Risk and resilience in the late glacial: a case study from the western Mediterranean. Quat. Sci. Rev..

[bib36] Belknap D.F., Kraft J.C. (1981). Preservation potential of transgressive coastal lithosomes on the U.S. Atlantic Shelf. Mar. Geol..

[bib37] Berger W.H., Yasuda M.-K., Bickert T., Wefer G., Takayama T. (1994). Quaternary time scale for ontong java plateau: milankovitch template for ocean drilling program site 806. Geology.

[bib38] Björck S., Walker M.J.C., Cwynar L., Johnsen S.J., Knudsen K.L., Lowe J.J., Wohlfarth B., Members Intimate (1998). An event stratigraphy for the Last Termination in the north Atlantic based on the Greenland Ice Core record: a proposal by the INTIMATE group. J. Quat. Sci..

[bib40] Breuil H., Burkitt M.C. (1929).

[bib41] Brown K., Fa D., Finlayson G., Finlayson Cl., Bicho N., Haws J. (2011). Trekking the Shore: Changing Coastlines and the Antiquity of Coastal Settlement. Interdisciplinary Contribution to Archaeology.

[bib42] Cabello L., Cantalejo P., Espejo Ma.M., Buendía A.F., Schmidt I., Castaleira J., Bicho N., Weniger G.-C. (2019). Human Adaptatios to the Last Glacial Maximun. The Solutrean and its Neighbors.

[bib43] Cacho I., Grimalt J.O., Pelejero C., Canals M., Sierro F.J., Flores J.A., Shackleton N. (1999). Dansgaard-oeschger and heinrich event imprints in Alboran Sea paleotemperatures. Paleoceanography.

[bib44] Cacho I., Grimalt J.O., Canals M., Sbaffi l., Shackleton N.J., Schönfeld J., Zahn R. (2001). Variability of the western Mediterranean Sea surface temperature during the last 25.000 years and its connection with the Northern Hemisphere climate changes. Paleoceanography.

[bib45] Camuera J., Jiménez-Moreno G., Ramos-Román Ma.J., García-Alix A., Toney J.L., Scott Anderson R., Jiménez-Espejo F., Bright J., Webster C., Yanes Y., Carrión J.S. (2019). Vegetation and climate changes during the last two glacial-interglacial cycles in the western Mediterranean: a new long pollen record from Padul (southern Iberian Peninsula). Quat. Sci. Rev..

[bib46] Cantalejo P. (1983). La cueva de Malalmuerzo (Moclín, Granada): nueva estación con Arte Rupestre Paleolítico en el área mediterránea. Antropol. Paleoecol. humana.

[bib47] Cantalejo P., Espejo M.M. (2014).

[bib48] Cantalejo P., Espejo Ma.M., Maura R., Ramos J.F., Aranda A. (2006).

[bib49] Cantalejo P., Maura R., Aranda A., Espejo M.M., La Serranía (2007). Málaga.

[bib50] Carranza J., Salvador A., Cassinello J. (2011). Enciclopedia Virtual de los Vertebrados Españoles.

[bib51] Collado H., Bea M., Ramos J., Cantalejo P. (2019). Un nuevo grupo de manos paleolíticas pintadas en el sur de la Península Ibérica: la Cueva de las Estrellas (Castellar de la Frontera, Cádiz). Zephyrus.

[bib52] Collado H., Fernandez D.S., Ramos J., Vijande E., Luque A., Dominguez-Bella S., Cantillo J.J., Montañés M., Bea M., Angás J., García-Arranz J.J., Carrascal J.M., Mira H., Escalona S. (2020). Nuevos motivos de manos paleolíticas en la Cueva de las Palomas IV de Facinas (Tarifa, Cádiz). Almoraima. Rev. Estud. Campogibralt..

[bib53] Colonese A.C., Mannino M.A., Bar-Yosef Mayer D.E., Fa D.A., Finlayson J.C., Lubell D., Stiner M.C. (2011). Marine mollusc exploitation in Mediterranean prehistory: an overview. Quat. Int..

[bib55] Cruz J.A., Hernández-Molina F.J., Vázquez J.T. (1992).

[bib56] Cunnane S.C., Stewart K.M. (2010). Human Brain Evolution: the Influence of Freshwater and Marine Food Resources.

[bib57] Davidson I. (1973). The fauna from la Cueva del Volcán del Faro (Cullera, Valencia). Arch. Prehist. Levantina.

[bib58] Doadrio I. (2002). Atlas y Libro Rojo de los Peces Continentales de España.

[bib59] Duane D.F., Field M.E., Meisburger E., Swift D.J.P., Williams S.J., Swift D.J.P., Duane D.B., Pilkey O.H. (1972). Shelf Sediment Transport: Process and Pattern. Dowden, Hutchinson and Ross, Strodsburg.

[bib60] Durán J.J., Soria J.M. (1989).

[bib61] Durán Valsero J.J. (1996).

[bib62] Eastham A., Jordá Pardo J.F. (1986). La Prehistoria de la Cueva de Nerja, Patronato de la Cueva de Nerja.

[bib63] Erlandson J.M. (2001). The Archaeology of aquatic adaptations: paradigms for a new millennium. J. Archaeol. Res..

[bib64] Espejo M.M., Cantalejo P. (1996). Arte prehistórico en las cuevas del Cantal, Rincón de la Victoria (Málaga). Rev. Arqueol..

[bib65] Fernández D.S., Ramos J., Collado H., Vijande E., Luque A.J. (2019).

[bib66] Fernández Salas L.M. (1996).

[bib67] Fernández-López de Pablo J., Gómez-Puche M., Martinez-Ortí A. (2011). Systematic consumption of non-marine gastropods at open-air Mesolithic sites in the Iberian Mediterranean región. Quat. Int..

[bib68] Fernández-Salas L.M. (2008).

[bib69] Fernández-Salas L.M., Durán R., Mendes I., Galparsoro I., Lobo F.J., Bárcenas P., Rosa F., Ribó M., García-Gil S., Ferrín A., Carrara G., Roque C., Canals M. (2015). Shelves of the iberian Peninsula and the balearic islands (I): morphology and sediment types. Bol. Geol. Min..

[bib70] Ferré E., Cortés M., Ramos J., Senciales J.M., Lozano-Francisco M.C., Vera-Peláez J.L., Aguilera R., Navarrete I. (2003). El Cuaternario reciente en el sector oriental de la bahía de Málaga. Rasas y depósitos marinos, continentales y arqueológicos. Cuaternario Geomorfol..

[bib71] Ferrer J.E., Marqués I., Cortés M., Ramos J., Baldomero A., Sanchidrián J.L., Márquez A.M., Fullola J.M. (2006). La Cuenca Mediterránea durante el Paleolítico Superior (38.000 – 10.000 años).

[bib72] Finlayson C., Giles-Pacheco F., Rodríguez-Vidal J., Fa D., Gutiérrez J.M., Santiago A., Finlayson G., Allué E., Baena J., Cáceres I., Carrión J.S., Fernández- Jalvo Y., Gleed-Owen C.P., Jiménez-Espejo F., López P., López-Sáez J.A., Riquelme J.A., Sánchez-Marco A., Giles-Guzmán F., Brown K., Fuentes N., Valarino C., Villalpando A., Stringer C.B., Martínez-Ruíz F., Sakamoto T. (2006). Late survival of Neanderthals at the southernmost extreme of Europe. Nature.

[bib73] Flemming N.C. (1972). Relative chronology of submerged Pleistocene marine erosion features in the western mediterranean. J. Geol..

[bib74] Fortea F.J. (1973).

[bib75] Fortea F.J. (1986). Actas del Congreso Homenaje a L. Siret.

[bib76] Fortea F.J., Giménez M. (1973). La Cueva del Toro. Nueva estación malagueña con Arte Paleolítico. Zephyrus.

[bib77] García-Argudo G. (2018). Mangado, X. (coord.): Montlleó: el paleolític superior a la Cerdanya. Resultats de 20 anys de recerca arqueológica. Homenatge a Oriol Mercadal Fernàndez.

[bib78] Giménez Reyna S. (1946).

[bib79] Gofas S., Moreno D., Salas C. (2011).

[bib80] Gofas S., Moreno D., Salas C. (2011).

[bib81] Gordón J.J. (2017). Interaccions entre felins i humans. III Jornades d’arqueozoologia.

[bib82] Granados J.E., Serrano E., Pérez C., Fandos P., Stefanie W., Soriguer R.C., Jiménez Pérez, M J. (2004). Memoriam Al Prof. Dr. Isidoro Ruiz Martínez.

[bib83] Grootes P.M., Stuiver M., White J.W.C., Johnsen S., Jouzel J. (1993). Comparison of oxygen isotope records from the GISP2 and GRIP Greenland ice core. Nature.

[bib84] Gruet Y., Laporte L. (1996). Crabes peches au Neolithique final a Ponthezieres (Saint-Georges d’Oléon, Charente-Maritime): identifications, modes de pêche et applications de la métrique. Revue d’Archéométr..

[bib85] Hernández-Molina F.J. (1993).

[bib86] Hernández-Molina F.J., Vázquez J.T., de la Cruz J.A., Rey J., Somoza L., Medialdea T., San Gil C., Díaz del Río V., Maldonado A. (1992). El Mar de Alborán y el Golfo de Cádiz: Conexiones Atlántico-Mediterráneo, III Congreso Geológico de España, Salamanca.

[bib87] Hernández-Molina F.J., Vázquez J.T., Somoza L., Rey J. (1993). Estructuración sedimentaria de los cuerpos deltaicos Holocenos del margen septentrional del Mar de Alborán. Geogaceta.

[bib88] Hernández-Molina F.J., Gracia F.J., Somoza L., Rey J., Arnáez J., García Ruiz A., Villar G. (1994). Geomorfología en España, III Reunión de Geomorfología.

[bib89] Hernández-Molina F.J., Somoza L., Rey J., Pomar L. (1994). Late Pleistocene-Holocene sediments on the Spanish continental shelves: model for very high resolution sequence stratigraphy. Mar. Geol..

[bib90] Hernández-Molina F.J., Somoza L., Vázquez J.T., Rey J. (1995). Estructuración de los prismas litorales del Cabo de Gata: respuesta a los cambios climático-eustáticos holocenos. Geogaceta.

[bib91] Hernández-Molina F.J., Gracia F.J., Somoza L., Rey J. (1996). Distribución batimétrica de las terrazas submarinas en la plataforma continental de Málaga-Gibraltar. Implicaciones eustáticas durante el Cuaternario terminal. Geogaceta.

[bib92] Hillson S. (1996).

[bib93] Hoyo J.D., Elliott B., Christie D.A., SArgatal J.E. (1992-2010).

[bib94] Inizian M.-L., Tixier J., Roche H., Reduron-Ballinger M. (1995).

[bib95] Jerardino A., Parkington J. (1993). New evidence for whales on archeological sites in the south-western Cape. South Afr. J. Sci..

[bib96] Jordá Pardo J.F. (1981). La malacofauna de la cueva de Nerja (I). Zephyrus.

[bib97] Jordá Pardo J.F. (1982). La malacofauna de la cueva de Nerja (II): los elementos ornamentales. Zephyrus.

[bib98] Jordá Pardo J.F., Jordá F.J. (1986). La Prehistoria de la Cueva de Nerja.

[bib99] Jordá Pardo J.F., Aura J.E., Martín C., Avezuela B., Álvarez-Fernández E., Carvajal-Contreras D.R. (2010). Not Only Food. Marine, Terrestrial and Freshwater Molluscs in Archaeological Sites.

[bib100] Jorda Pardo J.F., Maestro A., Aura J.E., Álvarez-Fernández E., Avezuela B., Badal E., Morales J.V., Pérez-Ripoll M., Villalba Ma.P. (2011). Evolución paleográfica, paleoclimática y paleoambiental de la costa meridional de la Península Ibérica durante el Pleistoceno superior. El caso de la Cueva de Nerja (Málaga, Andalucía, España). Boletín de la Real Sociedad Española de Historia Natatural. Secc. Geol..

[bib101] Jordá Pardo J.F., Aura J.E., Avezuela B., Álvarez-Fernández E., García-Pérez A., Maestro A. (2016). Breaking the waves. Human use of marine bivalves in a microtidal range coast during the Upper Pleistocene and the Early Holocene: the case of Nerja Cave (Málaga, southern Spain). Quat. Int..

[bib102] Kandel A.W., Conard N.J. (2003). Scavenging and progressing of the whale mear and blubber by later snote age people of the geelbel dunes, western cape province. S. Afr. Archaeol. Bull..

[bib103] Lario J., Zazo C., Somoza L., Goy J.L., Hoyos M., Silva P.G., Hernández F.J. (1993). Los episodios marinos cuaternarios de la costa de Málaga (España). Rev. Soc. Geol. Espana.

[bib104] Lario C.J., Zazo C., Goy L.J., Hoyos M., Hillaire Marcel C., Senciales J.M., Ferre E., coords (1998). Elementos de los paisajes de la provincia de Málaga.

[bib105] Lavocat R. (1966).

[bib106] Le Gall O. (1984).

[bib107] Lhénaff R. (1967).

[bib108] Llopis Llado N. (1970).

[bib109] Lloveras Ll., Moreno-García M., Nadal J., Zilhão J. (2011). Who brought in the rabbits? Taphonomical analysis of mousterian and solutrean leporid accumulations from gruta do caldeirão (tomar, Portugal). J. Archaeol. Sci..

[bib110] Lloveras Ll., Maroto J., Soler J., Thomas R., Moreno-García M., Nadal J., Soler N. (2016). The role of small prey in human subsistence strategies from Early Upper Palaeolithic sites in Iberia: the rabbits from the Evolved Aurignacian level of Arbreda Cave. J. Quat. Sci..

[bib111] Lobo F.J., Fernández-Salas L.M., Moreno I., Sanz J.L., Maldonado A. (2006). The seafloor morphology of a Mediterranean shelf fed by small rivers, northern Alboran Sea margin. Continent. Shelf Res..

[bib112] Maestro A., López-Martínez J., Llave E., Bohoyo F., Acosta J., Hernández-Molina F.J., Muñoz A., Jané G. (2013). Geomorphology of the iberian continental margin. Geomorphology.

[bib113] Marean C.M. (2014). The origins and significance of coastal resource use in Africa and Western Eurasia. J. Hum. Evol..

[bib114] Marean C.W., Bartthews M., Bernatchez J., Fisher E., Goldberg P., Herries A.I.R., Jacobs Z., Jerardino A., Karkanas P., Minichillo T., Nilssen P.J., Thompsom E., Watt I., Williams H.M. (2007). Early human use of marine resources and pigment in South Africa during the Middle Pleistocene. Nature.

[bib115] Márquez Alcántara A.M., Sanchidrián Torti J.L., Martínez García J., Hernández Pérez M.S. (2006). Actas del Congreso de Arte Esquemático en la Península Ibérica (Comarca de los Vélez, 5-7 de mayo 2004).

[bib116] Martínez S.V. (2015).

[bib117] Martínez Andreu M. (1989).

[bib118] Medved I. (2013).

[bib119] Meese D., Alley R., Gow T., Grootes P.M., Mayewski P., Ram M., Taylor K., Waddington E., Zielinski G. (1994).

[bib120] Mestres J.S. (1995). La datació per radiocarboni i el calibratge de les dates radiocabòniques. Objectius, problemes i aplicacions. Rev. d’Arqueol. Ponent.

[bib121] Mestres J.S. (2000). Tribuna d’Arqueologia 1997-1998.

[bib122] Mestres J.S. (2003). La química i la cronologia: la datació per radiocarboni. Rev. Soc. Catal. Quím..

[bib123] Mestres J.S. (2008). El temps a la Prehistòria i el seu establiment a través de la datación per radiocarboni. Cypsela.

[bib124] Morales Pérez J.V., Alcover J.A., Jordá Pardo J.F., Aura J.E. (2020). Tafonomía, Taxonomía, Paleoclimatología Y Contextualización Arqueológica. Libro Homenaje Al Prof. M. Pérez Ripoll.

[bib125] Morales-Pérez J.V. (2015).

[bib126] Moreno R. (1994).

[bib127] Nadihuska Y., Rosado-Méndez N.Y., Lloveras Ll., García-Argüelles G.A., Nadal J. (2018). The role of small prey in hunter–gatherer subsistence strategies from the Late Pleistocene–Early Holocene transition site in NE Iberia: the leporid accumulation from the Epipalaeolithic level of Balma del Gai site. Archaeol. Anthropol. Sci..

[bib128] North Greenland Ice Core Project (2004). High-resolution record of Northern Hemisphere climate extending into the last interglacial period. Nature.

[bib129] Pagel M.D., May R.M., Collie A.R. (1991). Ecological aspects of the geographical distribution and diversity of mammalian species. Am. Nat..

[bib130] Pales L., Garcia A. (1981).

[bib131] Pelegrin J. (2000). L'Europe Centrale et septentrionale au Tardiglaciaire.

[bib132] Pérez Ripoll M., Martínez R., Villaverde V. (2001). De Neandertales a Cromañones. El inicio del poblamiento humano en tierras valencianas.

[bib133] Pérez-Ripoll M., Brugal J.-F., Desse J. (2004). Petits animaux et societies humaines. XXIV Rencontres Intenationales d’Archéologie et d’Histoire d’Antibes. Centre d’Études Préhistoire, Antiquité, Moyen Âge, Ville d’Antibes.

[bib134] Pérez-Ripoll M., Martinez R. (1995). Análisis arqueozoológico de los restos. El Tossal de la Roca (Vall d’Alcalà, Alicante). Reconstrucción paleoambiental y cultural de la transición del Tardiglaciar al Holoceno inicial. Recerq. Museu. d’Alcoi..

[bib135] Pérez-Ripoll, Aura J.E., Jordá J., Morales J.V., Badal E., Sanchis A., Arribas A., Wood R. (2011).

[bib136] Perlès C. (1991). XI Rencontres Internationales, d'Archeologie et d'Historie d'Antibes “25 ans d'études technologiques en préhistoire.

[bib137] Ramos Fernández J. (2003). Actas de las jornadas temáticas andaluzas de arqueología, Ronda.

[bib138] Ramos Fernández J., Bañares M., Lozano Ma C., Vera J.L., Bicho N. (2006). Simbolismo, Arte e Espaços Sagrados na Pré-história da Península Ibérica. Centro de Estudos de Património. Departamento de História.

[bib140] Reimer P.J., Austin W.E.N., Bard E., Bayliss A., Blackwell P.G., Ramsey C.B., Butzin M., Cheng H., Edwards R.L., Friedrich M., Grootes P.M., Guilderson T.P., Hajdas I., Heaton T.J., Hogg A.G., Hughen K.A., Kromer B., Manning S.W., Muscheler R., Palmer J.G., Pearson C., van der Plicht J., Reimer R.W., Richards D.A., Scott E.M., Southon R., Turney C.S.M., Wacker L., Adolphi F., Büntgen U., Capano M., Fahrni S.M., Fogtmann-Schulz A., Friedrich R., Köhler P., Kudsk P., Miyake F., Olsen J., Reinig F., Sakamoto M., Sookdeo A., Talamo S. (2020). The IntCal20 Northern Hemisphere radiocarbon age calibration curve (0–55 cal kBP). Radiocarbon.

[bib141] Ripoll Perelló E. (1970).

[bib142] Rofes J., Zuluaga M.C., Murelaga X., Fernández-Eraso J., Bailon S., Iriarte-Chiapusso M.J., Ortega I.I., Alonso-Olazabal A. (2013). Paleoenvironmental reconstruction of the early neolithic to the middle bronze age at peña larga rock shelter (alava, Spain) from yhe small mammals record. Quat. Res..

[bib143] Román D., Martínez-Andreu M., Aguilella G., Fullola J.M., Nadal J. (2020). Shellfish collectors on the seashore: the exploitation of the marine environment between the end of the Paleolithic and the Mesolithic in the Mediterranean Iberia. J. I. Coast Archaeol..

[bib144] Rubio A. (1975). Las pinturas rupestres de la Cueva de la Victoria (La Cala, Málaga). Zephyrus.

[bib145] Ruddiman W., McIntyre A. (1981). the north Atlantic Ocean during the last deglaciation. Palaeogeogr. Palaeoclimatol. Palaeoecol..

[bib146] Sanchidrián J.L. (1982).

[bib147] Sanchis A. (2012).

[bib148] Sangree J.B., Widmer J.M., Payton C.E. (1977). Seismic Stratigraphy-Applications to Hydrocarbon Explorations.

[bib149] Schmid E. (1972).

[bib150] Schweingruber F. (1990).

[bib151] Sesé C., Montes R., Lasheras J.A. (2005). Neandertales Cantábricos, estado de la cuestión.

[bib152] Shakelton N.J. (1987). Oxygen isotopes, ice volume and sea level. Quat. Sci. Rev..

[bib153] Sonneville-Bordes D. (1960).

[bib154] Soriguer R.C. (1983). XV Congr. Int. Fauna Cinegética Y Silvestre, Trujillo.

[bib155] Southward A.J. (2008).

[bib156] Speybroek J., Beukema W., Crochet P.M. (2010).

[bib157] Such M. (1920). Avance al estudio de la caverna de “Hoyo de la Mina” en Málaga. Bolet. Soc. Malag. Cien..

[bib158] Svenning J.-Ch., Pedersen P.B., Donlan C.J., Ejrnæs R., Faurby S., Galetti M., Hansen D.M., Brody Sandel B., Sandom Ch.J., Terborgh J.W., Vera F.W. (2016). Science for a wilder Anthropocene: synthesis and future directions for trophic rewilding research. Proc. Natl. Acad. Sci. Unit. States Am..

[bib159] Tattersall I. (2014). Diet as driver and constraint in human evolution. J. Hum. Evol..

[bib160] Thompson W.G., Goldstein S.L. (2006). A radiometric calibration of the SPECMAP timescale. Quat. Sci. Rev..

[bib161] Tutin T.G., Heywood V.H., Burges N.A., Valentine D.H., Walters S.M., Webb D.A. (1964).

[bib162] Ungar P.S., Grine F.E., Teaford M.F. (2006). Diet in early Homo: a review of the evidence and a new model of adaptive versatility. Annu. Rev. Anthropol..

[bib163] Vadillo M. (2018).

[bib164] Vadillo M., Jardón P., Aura J.E. (2019). Los cantos epipaleolíticos de Coves de Santa Maira (Alicante): estudio funcional a partir de las marcas de uso y de la experimentación. Zephyrus.

[bib165] Vera J.A., Martín-Algarra A., Vera J.A. (2004). Geología de España.

[bib166] Vernet J.L., Ogereau P., Figueiral I., Machado C., Uzquiano P. (2001).

[bib167] Villaverde V., Roman D., Pérez Ripoll M., Bergadà M.M., Real C. (2012). The end of the upper paleolithic in the mediterranean basin of the iberian Peninsula. Quat. Int..

[bib168] Villaverde V., Aura J.E., Borao M., Román D., Langley M.C. (2016). Osseous Projectile Weaponry: towards an Understanding of Pleistocene Cultural Variability.

[bib169] Wang Y.J., Cheng H., Edwards R.L., An Z.S., Wu J.Y., Shen C.C., Dorale J.A. (2001). A high-resolution absolute-dated late Pleistocene monsoon record from Hulu cave, China. Science.

[bib170] Welter-Schultes F. (2012).

[bib171] Weninger B., Jöris O., Higham T., Bronk Ramsey C., Owen C. (2004). Radiocarbon and Archaeology.

[bib172] Weninger B., Jöris O. (2008). A 14C age calibration curve for the last 60 ka: the Greenland-Hulu U/Th timescale and its impact on understanding the Middle to Upper Paleolithic transition in Western Eurasia. J. Hum. Evol..

[bib173] WoRMS Editorial Board (2019).

[bib174] Zariquiey-Álvarez R. (1968). era 32). Barcelona.

[bib175] Zilhão J., Angelucci D.E., Araújo Igreja M., Arnold L.J., Badal E., Callapez P., Cardoso J.L., d’Errico F., Daura J., Demuro M., Deschamps M., Dupont C., Gabriel S., Hoffmann D.L., Legoinha P., Matias H., Monge Soares A.M., Nabais M., Portela P., Queffelec A., Rodrigues F., Souto P. (2020). Last interglacial iberian neandertals as Fisher-hunter-gatherers. Science.

